# The Encyclopedia of Proteome Dynamics: a big data ecosystem for (prote)omics

**DOI:** 10.1093/nar/gkx807

**Published:** 2017-09-07

**Authors:** Alejandro Brenes, Vackar Afzal, Robert Kent, Angus I Lamond

**Affiliations:** Centre for Gene Regulation and Expression, School of Life Sciences, University of Dundee, Dow St, Dundee DD1 5EH, UK

## Abstract

Driven by improvements in speed and resolution of mass spectrometers (MS), the field of proteomics, which involves the large-scale detection and analysis of proteins in cells, tissues and organisms, continues to expand in scale and complexity. There is a resulting growth in datasets of both raw MS files and processed peptide and protein identifications. MS-based proteomics technology is also used increasingly to measure additional protein properties affecting cellular function and disease mechanisms, including post-translational modifications, protein–protein interactions, subcellular and tissue distributions. Consequently, biologists and clinicians need innovative tools to conveniently analyse, visualize and explore such large, complex proteomics data and to integrate it with genomics and other related large-scale datasets. We have created the Encyclopedia of Proteome Dynamics (EPD) to meet this need (https://peptracker.com/epd/). The EPD combines a polyglot persistent database and web-application that provides open access to integrated proteomics data for >30 000 proteins from published studies on human cells and model organisms. It is designed to provide a user-friendly interface, featuring graphical navigation with interactive visualizations that facilitate powerful data exploration in an intuitive manner. The EPD offers a flexible and scalable ecosystem to integrate proteomics data with genomics information, RNA expression and other related, large-scale datasets.

## INTRODUCTION

Mass spectrometry (MS) - based proteomics is now the predominant approach used for large-scale detection and quantitation of proteins in cells and tissues (1). There has been a rapid growth in the number and size of proteomics datasets generated. This has been aided by the continual improvement of mass spectrometry instrumentation, featuring increased speed and resolution, combined with improvements in sample handling workflows and peptide chromatography.

Additional levels of complexity result from the multidimensional and dynamic nature of cell proteomes. Modern proteomics methods allow measurements of the many properties of proteins that are critical for their respective functions and contributions to cell phenotypes and disease mechanisms. This includes measurement of the kinetics of change in abundance and other protein properties in cells responding to perturbations, such as either physiological stimuli, or drug treatments etc.

There is a need to integrate new large, multi-dimensional proteomics datasets with both previous proteomics studies and with many other forms of large-scale datasets in the public domain. This includes other forms of ‘omics’ data, e.g. genomics and transcriptomics data and potentially also different types of data, such as healthcare records. To address these challenges, we have created the Encyclopedia of Proteome Dynamics (EPD), which was developed using our high-throughput, curated proteomics datasets, annotated with consistent metadata, using carefully controlled vocabulary.

The EPD was first introduced in 2013 (2). Based on user feedback and to meet the challenges of growing data volumes and complexity, as well as to provide improved scalability and additional interactive features, a major redesign of the EPD was undertaken. The new EPD was first released in March 2015 (http://www.peptracker.com/epd). This new version makes use of scalable noSQL (not only Structured Query Language) solutions combined with a client side JS (Java Script) library to visualise and analyse data interactively. It is a unified online resource that combines processed MS-based proteomics data from many large-scale studies on multiple human cell types and model organisms obtained from the Lamond laboratory and collaborators (https://peptracker.com/epd/collaborators). It includes data from many different types of proteomics experiments, e.g. biological responses, cell cycle, as well as other types of data, e.g. RNAseq. For the past year it has had a median of over 18 457 hits and 698 unique users per day. The EPD is part of the PepTracker software suite, which includes other tools designed for convenient laboratory data management, metadata tracking and data analysis.

This article focusses on the design, functionality and implementation of this redesigned version of the EPD, as currently available, including feature updates incorporated up to July 2017. We describe below the functionality currently implemented in the EPD, how it can be used and how the data model and infrastructure is applied flexibly to integrate diverse, large-scale datasets and link all these data conveniently with other related information in the public domain.

## ARCHITECTURE: SCALABILITY AND RESILIENCE

The EPD was designed as a big data solution, using a noSQL ecosystem. The EPD combines a graph database and a key-store columnar database hybrid, providing near linear data scalability and excellent query performance.

The EPD uses Neo4j as the graph engine to model and *cypher* as the language to query the hierarchies and associations. The key-store columnar hybrid used in the EPD is apache Cassandra (http://cassandra.apache.org/). It operates by dividing all data evenly across all the nodes in a ring architecture. It does not use a master slave methodology; as such there is no single point of failure in the system. When set up appropriately, a node can become temporarily unavailable, or even lost permanently, without affecting either the integrity of the cluster, or the underlying data, making the EPD fault tolerant and resilient.

Both of the EPD’s noSQL databases provide horizontal, rather than vertical scalability, a cornerstone of solutions oriented to *store and process* big data. Thus, the EPD uses a cluster of commodity servers to form the foundation of the ecosystem. When additional storage, processing and/or memory are required, more nodes can readily be added into the cluster, making it easy to scale and maintain performance.

The EPD also uses D3.js as the client side java script (JS) library, which was chosen for its flexibility in providing a wide variety of visualization types suitable for displaying data from a diverse range of experimental scenarios. We note that while many other JS libraries have a simpler programmatic access layer, most only offer a small set of predetermined visualizations, making them less flexible for the rapidly changing requirements in the EPD.

## DATA ACCESS AND NAVIGATION

The ProteomeXchange consortium provides a valuable community resource for open access sharing of published raw MS data files (3,4). Our vision in creating the EPD, however, was not to duplicate a raw MS data repository, but rather to create a user-friendly, online platform where quantified proteomics data and other related, large-scale datasets can be integrated, interactively explored and efficiently searched.

Therefore, the EPD was explicitly designed to facilitate the ability of investigators to search, explore, download and analyse published datasets. These data are freely available at (https://peptracker.com/epd/analytics), via both desktop and mobile devices. The login page is available at (https://peptracker.com/epd), by clicking on the ‘Enter’ button it provides access to all the public datasets.

However, the EPD also provides additional security options; for example, datasets have restricted access before publication. By accessing the EPD with a registered account and logging in with valid credentials, researchers working on projects still in progress can take advantage of the interactive EPD tools for conveniently exploring and analysing their newly acquired data, prior to publication and open access sharing.

**Figure 1.**
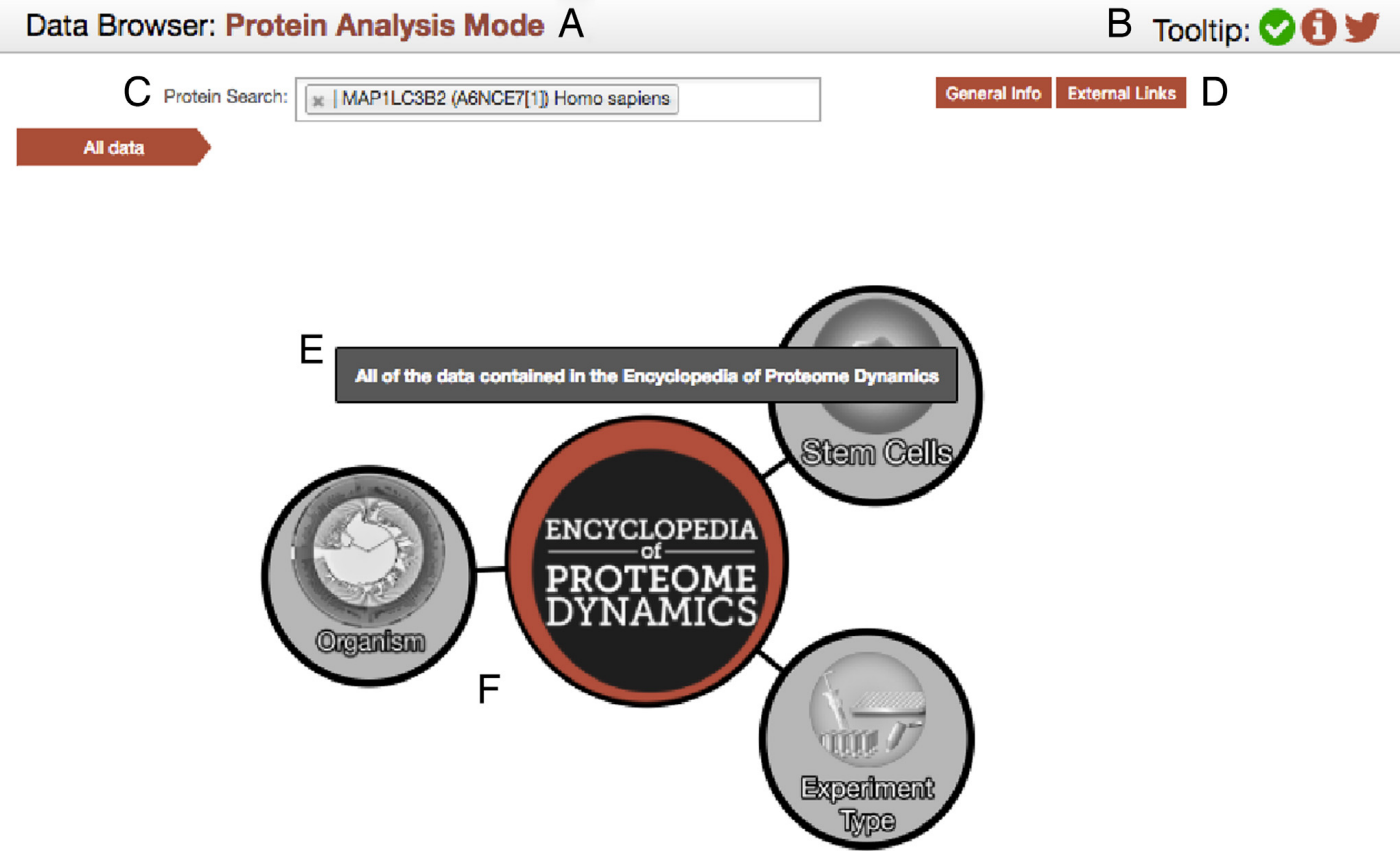
The graphical navigation element of the EPD. (**A**) The search mode display bar has two values; either protein analysis mode, or global analysis mode. The former filters out datasets that have not detected the selected protein, the latter allows access to all public datasets (and private ones if the user has the appropriate credentials). (**B**) The icons section: displays on the far right include a button with a link to the EPD’s twitter page, the middle button opens an information pop-up about the EPD and the left button a tooltip control. The tooltips can but toggled on/off by clicking on this button (represented by a white check inside a green circle). It is enabled on desktops and disabled on mobile/tablets by default. (**C**) The EPD search box: This input box is used to search for datasets containing a specific protein of interest. The search can be performed by typing either the gene name, protein description, protein name, or protein accession. It is automatically updated to search for protein association in the current hierarchy. (**D**) Information button: These two buttons are only visible for the protein analysis mode. They display additional information and external links for the selected protein. (**E**) The tool tip: This element is only visible if it has both been enabled (see B above) and the cursor is above a node in the force diagram. It provides a longer description of the element that is currently being hovered over. (**F**) The force diagram: This is the interactive graphical navigation element. It displays the current element in the hierarchy and allows the user to click on nodes to advance to the child nodes.

To provide users with convenient access to interact with the large and diverse repository of data it contains, the EPD has implemented an intuitive, graphical navigation interface, based on a directed force diagram and powered by the underlying graph database. The navigation is provided by an interactive component that displays a visual representation of the available elements, as represented in Figure 1. These components are in turn parts of different hierarchies. By mousing-over any of them, a tooltip will be displayed (if this option is enabled as described in Figure 1) that describes the current node. Once a node is clicked, it updates the displayed hierarchy to reveal its child elements.

This navigation model is flexible and not restricted to proteomics data. For example, the EPD already includes examples of projects integrating proteomics and cognate mRNA data (5). Work is currently in progress to incorporate other forms of ‘omics’ data, including genome sequence data. We note that the flexibility provided by the graph and the hierarchical structure are widely applicable to many diverse analytical datasets, which can include clinical and healthcare data.

**Figure 2.**
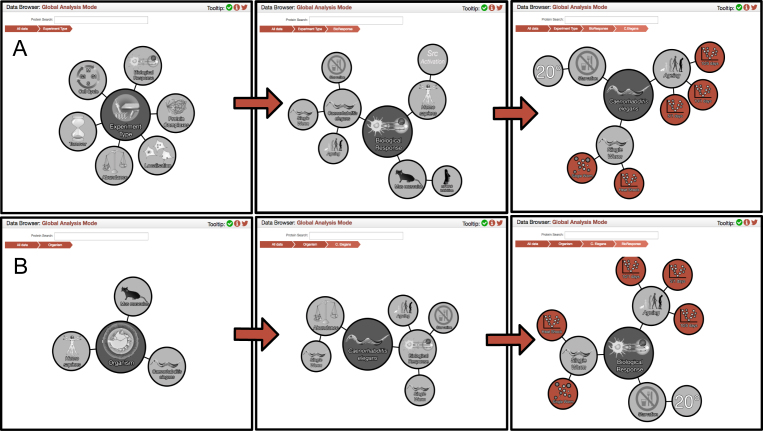
Navigating the hierarchies. (**A**) The different nodes within the ‘Experiment Type’ hierarchy that lead to nematode Biological Response data. (**B**) The different nodes within the ‘Organism’ hierarchy that lead to the same end-point as the former.

Amongst the first nodes that can be explored are ‘Experiment Type’ and ‘Organism’. Each of these nodes is the start, or root level, of a hierarchy that leads to the same collection of end nodes. This design allows users to navigate to their destination through a selected path that is most relevant and/or intuitive to their interests, as illustrated in Figure 2.

The hierarchies within the navigation provide contextual descriptions of how the different individual datasets relate to each other. To provide a visual representation of this, the top pane holds a breadcrumb trail that is automatically updated when the user clicks on any node. Akin to the file explorer visualization in both Windows and macOS, the EPD breadcrumb trail is used to display the hierarchical order of the elements that have been navigated and can also be used to navigate back to any part of the hierarchy that had been accessed previously, by directly clicking on that breadcrumb element in the top pane.

The EPD currently provides two main modes of use to explore the data, i.e. either *global* analysis mode, or *protein* analysis mode. The default *global* mode provides unfiltered access to all public datasets within the EPD. The *protein* analysis mode is activated by simply searching for a specific element, e.g. protein, selected from a drop-down menu, accessed via the search box. The search box provides access to a powerful search function, extensively using Neo4j's schema-indexed text search to provide an efficient and intelligent search. For example, a search can be initiated for a specific protein by entering either a gene name, protein description, protein name, or protein UniProt accession. As soon as more than 2 characters are typed into the search box, the EPD will start to suggest options that match the input, displayed via a dropdown menu. Once an element is selected, i.e. by clicking on it from the list provided, the protein analysis mode is activated. This mode is a filtered subset of the data available via the global analysis mode and is restricted to displaying results specific to the selected protein; thus, datasets where the protein selected in the search window has not been detected will not be displayed.

As of July 2017, the EPD contains open access information for a total of 30,946 distinct proteins, detected in multiple types of experiments performed using cells from human (5–9), mouse (10) and nematode (11–14). Access to these data, in addition to directly searching the EPD, can also be achieved via the UniProt resource (15). UniProt provides convenient links for all proteins that have been detected in public datasets within the EPD under their ‘proteomics data bases’ heading.

## DATA GROWTH, COMPLEXITY AND INTEGRATION

The EPD integrates many proteomics datasets from human, including data from multiple cell types, as well as data from the model organisms *Caenorhabditis elegans* and *Mus musculus*. Including both already published data and data waiting publication, the EPD currently stores >142 million peptide spectrum matches (PSM) and >590 000 distinct peptide sequences. In the last 18 months, the data volume has grown by ∼ 300%.

The process of integrating new data into the EPD begins using text files generated by the software chosen for the interpretation of the raw MS files. We use an Extraction Transformation and Loading (ETL) step to convert elements from the typical text file outputs obtained from raw MS file analysis, via Spark (https://spark.apache.org/), into analytical data stored in the EPD’s noSQL databases.

**Figure 3.**
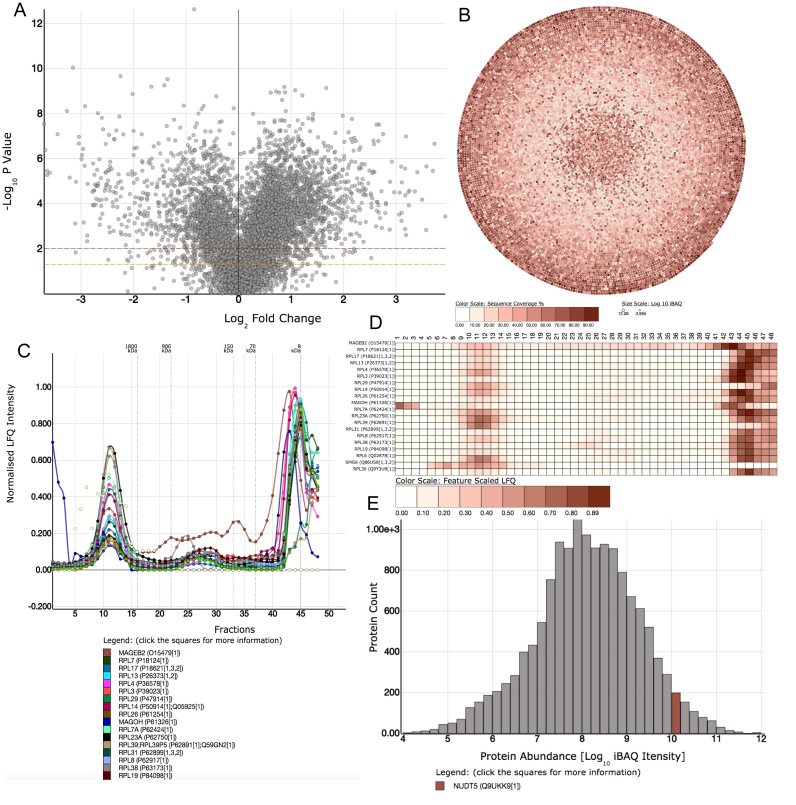
Examples of dynamic plots available in the EPD. (**A**) Volcano plot: a form of scatter plot used frequently in the EPD to represent changes in a proteome between conditions (e.g. drug treatment, time points etc.). (**B**) Bubble plot: showing the abundance and sequence coverage of every protein identified in the dataset. Data can be displayed in either log_10_ or raw abundance measures. (**C**) Line plot: used to display both elution profiles of protein complexes and for time course studies. (**D**) Heatmaps: Used to show elements that have been clustered together by machine learning algorithms, e.g. protein complex data. (**E**) Histogram: Used to show the log_10_ abundance of elements within the dataset. For example, when a protein is selected, it's corresponding bin is highlighted, quickly displaying the abundance category of the protein.

The graph database provides the model for data integration within the EPD. Thus, it holds the associations between experiments, datasets, proteins, genes, transcripts and chromosomes etc. Using this model, every new dataset becomes fully unified within the ecosystem and can be mapped and merged with all the pre-existing omics studies. To extend the utility of the data analysis functionality provided by the EPD, additional external data used for analysis on the plots are added. For example, the graph also holds annotations provided by the Gene Ontology (16), CORUM (17) and Reactome (18) databases. In addition, future plans include work in progress to integrate information on disease ontologies and drug targets. These external data elements are made available seamlessly to the user for data exploration and analysis within the EPD data visualizations. A detailed overview of the plot types and visualizations available in the EPD is provided in the Supplementary section and described also below (Figure 3).

## VISUALIZATION AND ANALYTICS

A key objective of the EPD is to provide convenient access for interactive exploration of large-scale, processed proteomics data and related information. At the simplest level, the EPD provides the user with the ability to filter and search all the datasets it contains, to display onscreen a visualization of any selected dataset and then, if required by the user, provide a one-click link to download a comma separated values (csv) file of the dataset selected. The EPD also provides a one-click link to download a scalable vector graphics (svg) file of the visualization displayed onscreen.

We have used graphical representations throughout the EPD user interface to aid navigation and filtering of the datasets. To maximise value by simplifying exploration and analysis of the data in the EPD, we don’t just provide access to large tables of data, but have designed highly interactive visualizations, using D3 as a client side Java Script (JS) library.

At the end point of each navigation hierarchy in the EPD lies one/multiple red node/s, representing a specific type/s of dynamic plot/s (interactive graphs). There are currently >100 different interactive visualizations available in the EPD, ranging from volcano plots to parallel coordinate plots. The type of visualization is selected to be the most appropriate for the class of information provided by each respective dataset. The interactive visualizations are launched by clicking on any red node, via the graphical navigation interface.

**Figure 4.**
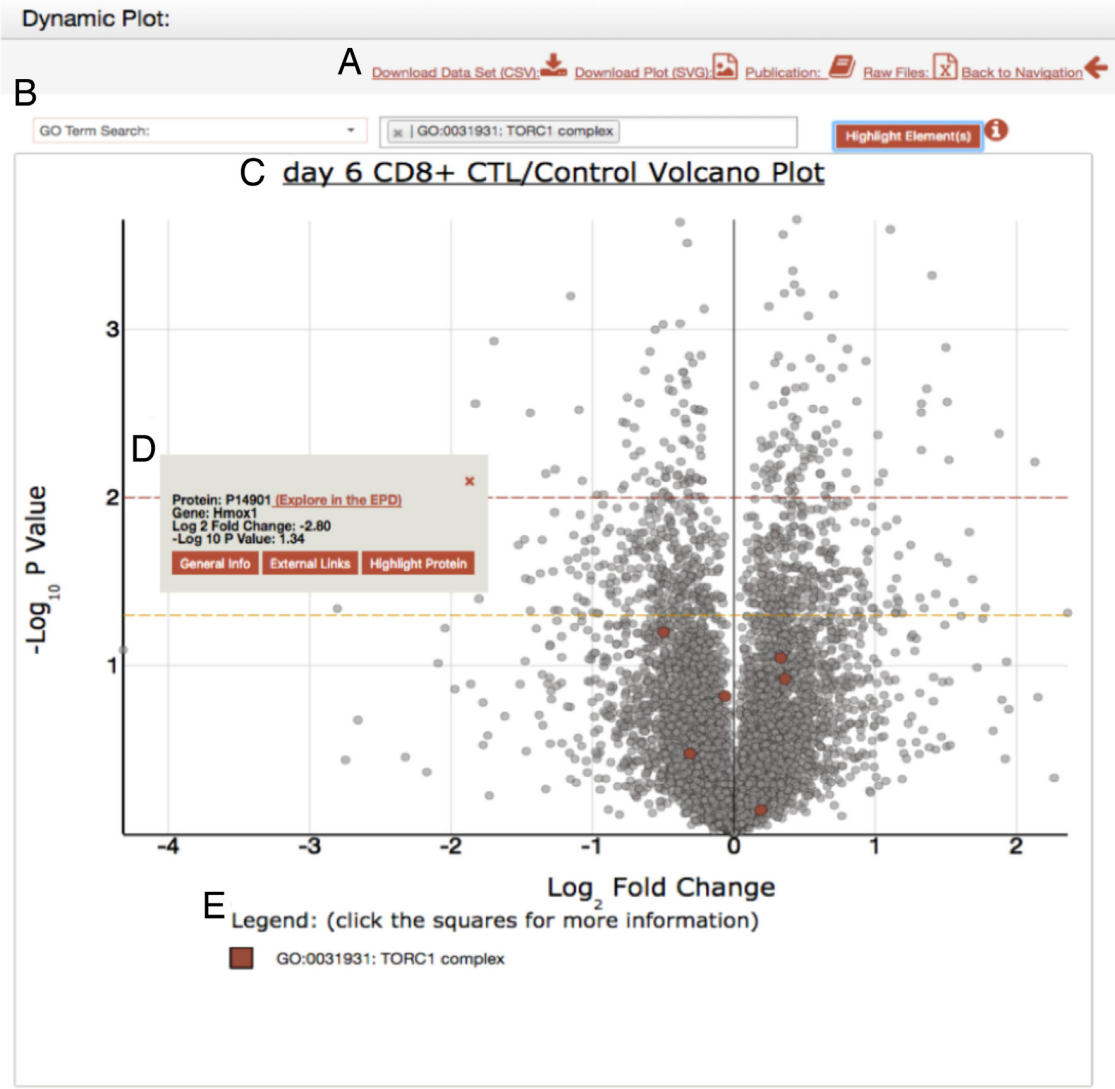
Example showing the EPD dynamic plot window. (**A**) The dataset functionality bar: This section allows a user to download either; the plot as a svg file, download the dataset as a csv file, or to access the relevant publication describing the data shown. Finally, a link is also provided to the PRIDE open access data repository that allows a user to download also the original raw MS files. (**B**) The search bar. The dropdown menu displays the different contextual elements integrated for search: currently either a GO Term, Protein complex, Reactome Pathway or a protein. (**C**) Example of a dynamic plot, in this case a volcano plot showing protein levels in mouse CD8+ cytotoxic T cells treated with rapamycin. The orange dotted line represents a *P*-value of 0.05 and the red line represent a *P*-value of 0.01. The *P*-values are calculated with a two-tailed *t* test and a Bonferroni correction. (**D**) The tooltip that is displayed when a click event is performed over any protein. This provides additional information about the specific element selected on the graph. (**E**) The legend displays the name(s) of the selected elements on the plot, along with a box demonstrating the color associated with the element.

Figure 3 shows some of the types of interactive visualizations that are available within the EPD (see Supplementary section for further descriptions of the interactive visualizations). For example, with datasets that compare how different biological conditions affect the proteome, a volcano plot would display the fold change in abundance of each protein on the x-axis and its corresponding statistical significance, calculated as a *P*-value from biological replicates, on the y-axis. Examples in the EPD include datasets comparing the proteomes of human cells at different cell cycle stages, comparing proteomes of nematodes that had either been fed, or starved, for specific time periods and comparing either stimulated, or unstimulated, control mouse T cells (see Figure 4).

Other forms of visualization are used to display data resulting from the analysis of protein complexes by Size Exclusion Chromatography (SEC)–MS. In this case a line plot showing the elution profiles of either one, or more, selected proteins across the different SEC fractions is displayed and either a heatmap, or force diagram, used to represent the elements that form the different clusters of co-eluting proteins in the chromatography experiments. For many datasets, multiple visualizations are available, each displaying a specific type of analysis, complemented by the interactive capabilities relevant to that plot (see Supplementary section for further details).

Each of the visualizations are implemented via the client side JS library, illustrated by an interactive scatter plot in Figure 4. In this graph, every dot represents a separate protein in the dataset. Users can click on any point to select it and activate a tooltip box that provides information identifying the protein, together with basic information, including its UniProt ID & gene, along with links to obtain additional information and to access relevant external resources. For example, the ‘External Links’ button provides a cross-reference to find information on the selected protein in either the UniProt, PDB (19) or String (20) databases. Also, by clicking on the ‘Highlight Protein’ button in the tooltip box, the selected protein is marked with a colored circle and a legend is created below the plot with a square of the same colour and labelled with the gene name and protein accession. The colored square can be clicked subsequently to access the same tooltip box again.

A powerful search feature available from the tooltip box is the ‘Explore in the EPD’ option; when clicked, this opens a new Data Browser in Protein Analysis Mode that has filtered the EPD to display specifically datasets that include the selected protein of interest. This feature is not limited to scatter plots. All the visualizations available in the EPD have interactive tooltips that provide added functionality, Table 1 illustrates the examples of dynamic plots that are described in the supplemental data section.

**Table 1.** Summary of plot types described in the supplemental data section

Plot type	Example dataset	Section number
Line Plot	Osteosarcoma cell protein complexes crosslinked with formaldehyde.	2
Volcano Plot	CD8+ T cells treated with rapamycin for 6 days	3
Parallel Coordinate Plot	Phosphorylation site analysis on a *pig-1* mutant *C.elegans* embryo	4
PTM Site Comparison Scatter Plot	Phosphorylation site comparison between a *pig-1* mutant *C.elegans* embryo and a Bristol N2 wild type.	5
Heatmaps and Force Diagrams	Osteosarcoma cell protein complexes crosslinked with formaldehyde.	6
Bar plots, Box plots and Histograms	Cell cycle arrest in human myeloid leukemia cells	7
Bubble plots	Human Induced Pluripotent Stem Cells (HipSci) project	8

## APPLICATION PROGRAMMING INTERFACE

The EPD also provides an Application Programing Interface (API) to query an aggregate list of peptides that have been detected in the datasets (https://peptracker.com/epd/rest/peptide_aggregates/). This list contains every peptide detected in the open access proteomics experiments recorded in the EPD, together with its lowest posterior error probability (PEP) value and its highest identification ‘Score’, as provided by the Andromeda search engine (21).

The PEP is the calculated probability that a*n individual* peptide identification may be false. It is a conditional probability, computed using Baye's theorem and dependent on the Andromeda score. As such, the PEP works as a quality control filter for peptide identifications. These peptide data are used by UniProt (22) as part of their proteomics data output.

To avoid the inflation of false discovery values, which is known to happen when multiple datasets that were quantified separately are subsequently integrated (23), we have calculated a Global False Discovery Rate (GFDR) for each peptide referenced in the EPD. This is possible because all the datasets were quantified using the same search engine, Andromeda, which is part of the MaxQuant (24) environment, hence their score calculation is the same for all elements. The GFDR is recalculated every time a new dataset from the same organism is integrated into the EPD and is therefore always up to date when queried via the API. Within the currently published datasets, there are 255 583 distinct *Homo sapiens* peptides with GFDR, 184 347 for *Caenorhabditis elegans* and 93 381 for *Mus musculus*. Table 2 shows the full list of peptides, filtered by organism and the URL where they can be accessed (as of July 2017).

**Table 2.** Peptide API URLs and details

URL	Organism	File type
https:peptracker.com/epd/rest/peptide_aggregates/?organism=musmusculus&filetype=json	Mouse	JSON
https:peptracker.com/epd/rest/peptide_aggregates/?organism=musmusculus&filetype=tsv	Mouse	TSV
https:peptracker.com/epd/rest/peptide_aggregates/?organism=homosapiens&filetype=json	Human	JSON
https:peptracker.com/epd/rest/peptide_aggregates/?organism=homosapiens&filetype=tsv	Human	TSV
https:peptracker.com/epd/rest/peptide_aggregates/?organism=caenorhabditiselegans&filetype=json	Nematode	JSON
https:peptracker.com/epd/rest/peptide_aggregates/?organism=caenorhabditiselegans&filetype=tsv	Nematode	TSV

## FUTURE DIRECTIONS

Along with a future roadmap for new features that are currently in development, the EPD already incorporates a series of additional features that have been developed in conjunction with new, unpublished datasets that have not yet been released for open access. For example, in collaboration with the Reactome team (http://reactome.org/), we plan to integrate the Reactome pathway diagrams with the EPD datasets. This will allow end users to perform powerful interactive analyses on the different pathways, with proteomics data overlaid on top to determine how the different protein subunits behaved under divergent conditions.

Another major new feature currently being developed for future release in the EPD involves providing a user friendly, effective element for each respective visualization to interactively display all the specific peptides detected by MS within the experiment, mapped onto the primary amino acid sequence of the protein selected in the plot. Beyond this, we will also incorporate in the EPD additional mRNA level data, linked with cognate protein analyses.

Future updates to the EPD will also provide interactive visualization tools to conveniently relate and display the protein isoforms and mRNAs detected in cells, with reference to chromosome maps, gene locations and DNA sequence variants. This will be used, for example, to integrate and display the extensive set of poly-omics DNA, mRNA and protein data recently generated from a wide range of human induced pluripotent stem cells that were created from healthy donors (25) (http://www.hipsci.org/).

## CONCLUSIONS

The EPD provides a user-friendly, open access big data solution for the integration, visualization and analysis of large, complex proteomics and related omics datasets. It is a polyglot persistent ecosystem with a web-app, built with scalability and resilience as key goals and specifically created to facilitate interactive data exploration and analysis using either desktop, or mobile devices.

The dynamic visualizations present in the EPD, combined with the integrated external databases, provide powerful new ways to analyse and extract information from omics datasets, while maintaining simplicity of use and a user-friendly interface.

Furthermore, the data model and interactive analytics platform can be adapted and applied also to many different types of biological and clinical data, providing a paradigm for open access sharing and integration of biological and biomedical big data.

## Supplementary Material

Supplementary Data
