# Supplementary material for: The Encyclopedia of Proteome Dynamics: a big data ecosystem for (prote)omics
Source: Nucleic Acids Res. 2017 Sep 7;46(Database issue):D1202–9. doi: 10.1093/nar/gkx807 (PMC5753345; doi:10.1093/nar/gkx807)
Supplement: Supplementary Data [file gkx807_supp.docx]

Supplemental Data

Section 1: Navigation

The Encyclopedia of Proteome Dynamics (EPD) can be accessed at <https://peptracker.com/epd> . This link directs the user to the login page, illustrated in Supplemental Figure 1. There is a public access section that does not require credentials and can be reached simply by clicking on the ‘Enter’ button at the right-hand side of the window. The public section in the EPD provides access to published datasets generated by the Lamond group and their collaborators over the past seven years. The ‘Lab Member Access’ section located lower on the window provides password restricted access to additional, as yet unpublished, datasets arising from projects and experiments that are still in progress, including collaborative projects. Login accounts are provided to registered users of the PepTracker software suite (see <https://peptracker.com/>).


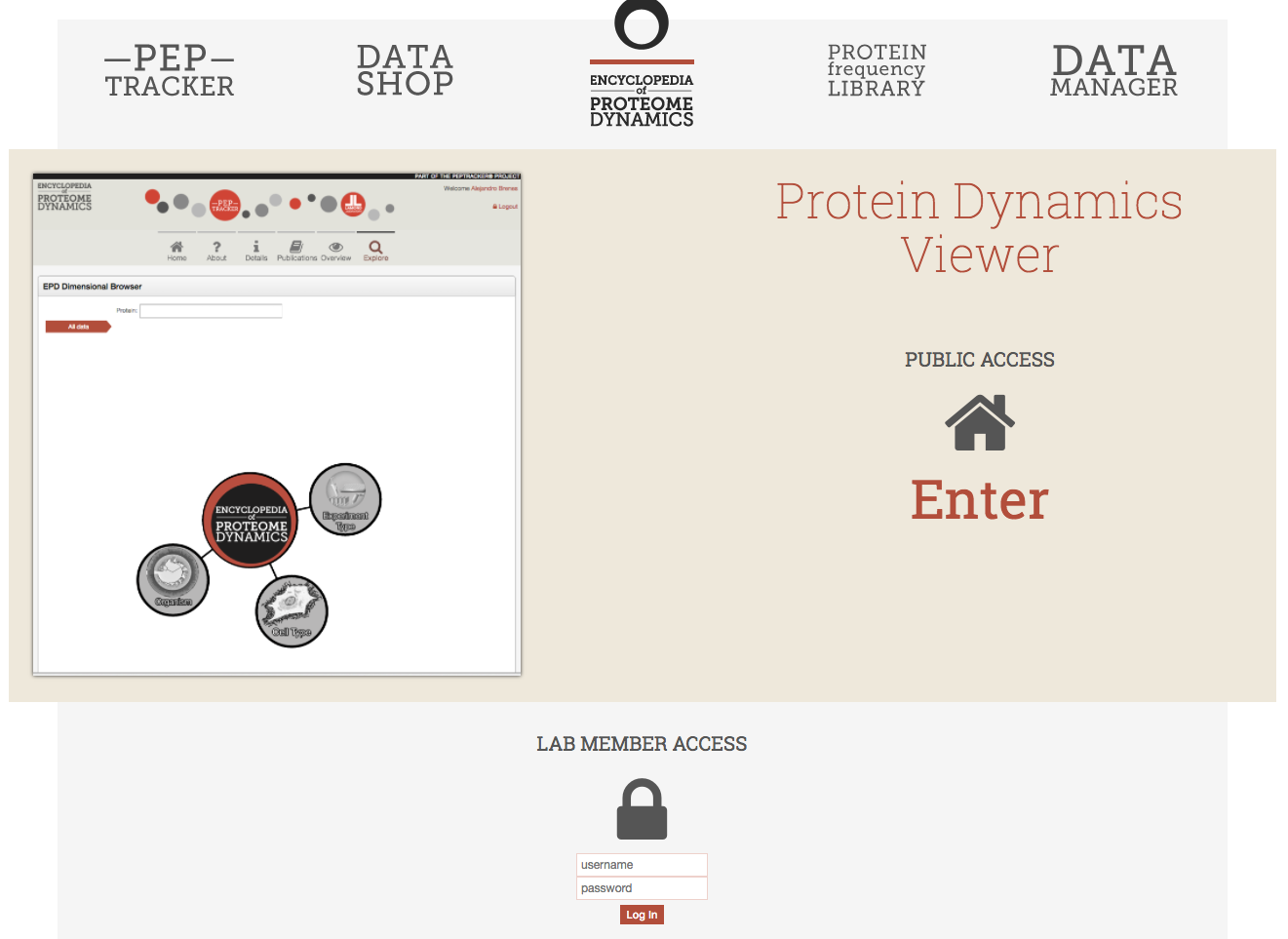


**Supplemental Figure 1:** The login page for the EPD.

After clicking on either the ‘Enter’ button, or providing login credentials, the user is redirected to the analytics section of the EPD. This an interactive element that provides a graphical interface for convenient navigation of the EPD datasets. The first nodes that can be explored represent the start, or root level, of a hierarchy that leads to the same collection of end nodes. For example, the root nodes currently include ‘Experiment Type’ and ‘Organism’. This design allows users to navigate to their destination through a selected path that is most relevant and/or intuitive to their interests, as illustrated in Supplemental Figure 2. Another root node is ‘Stem Cells’, which includes links to data arising from the Human Induced Pluripotent Stem Cells Initiative (HipSci) project (<http://www.hipsci.org/>).


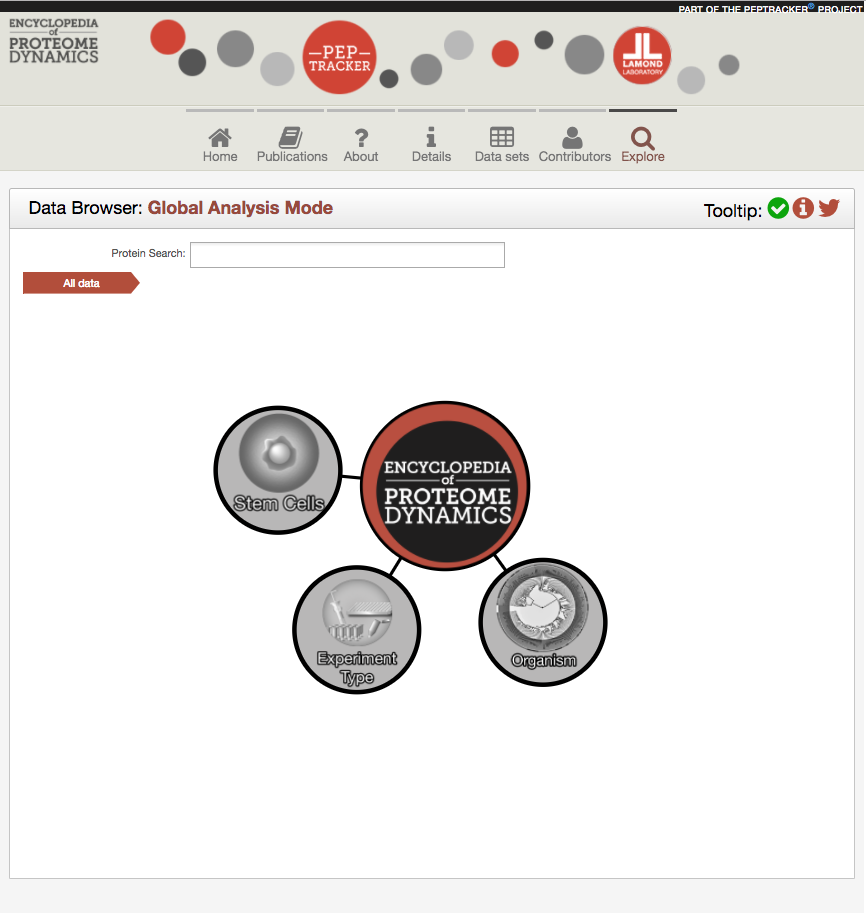


**Supplemental Figure 2:** The starting point for navigation of data available at <https://peptracker.com/epd/analytics>

The aforementioned nodes are all interactive elements. If the ‘Tooltips’ option is checked in the Data Browser bar at the top of the window (N.B. ‘Tooltips’ are active by default when viewing on a browser in a desktop/laptop and inactive by default when viewing on a browser in a mobile device), mousing over a node will reveal a tooltip that displays a description about the element. Clicking on a node will display their child nodes and thereby navigate through the hierarchy.

The hierarchies within the navigation provide contextual descriptions of how the different individual datasets relate to each other. To provide a visual representation of this, the top pane holds a breadcrumb trail that is automatically updated when the user clicks on any node. Akin to the file explorer visualisation in both Windows and macOS, the EPD breadcrumb trail is used to display the hierarchical order of the elements that have been navigated and can also be used to navigate back to any part of the hierarchy that had been accessed previously, by directly clicking on that breadcrumb element in the top pane.

**
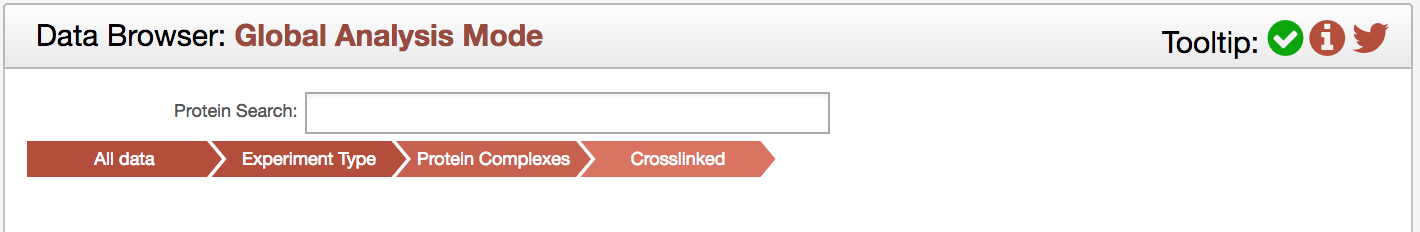
**

**Supplemental Figure 3:** The breadcrumb trail

The EPD currently provides two main modes of use to explore the data, i.e., either *global* analysis mode, or *protein* analysis mode. The default, *global* mode provides unfiltered access to all public datasets within the EPD. The *protein* analysis mode is activated by simply searching for a specific element, e.g. protein, selected from a drop-down menu, accessed via the search box, which is located at the top of the Data Exploration Window. The search box provides access to a powerful search function that makes extensive use of the schema-indexed text search functionality in Neo4j to provide an efficient and intelligent search. For example, a search can be initiated for a specific protein by entering either a gene name, protein description, protein name, or UniProt accession ID. As soon as more than 2 characters are typed into the search box, options will be displayed, via a dropdown menu, which match the search, as illustrated in Supplemental Figure 4. The protein analysis mode is activated once an element is selected by clicking on it from the list provided. This mode is a filtered subset of the data available via the global analysis mode and is restricted to displaying results specific to the selected protein; thus, datasets where the protein selected in the search window has not been detected will not be displayed.


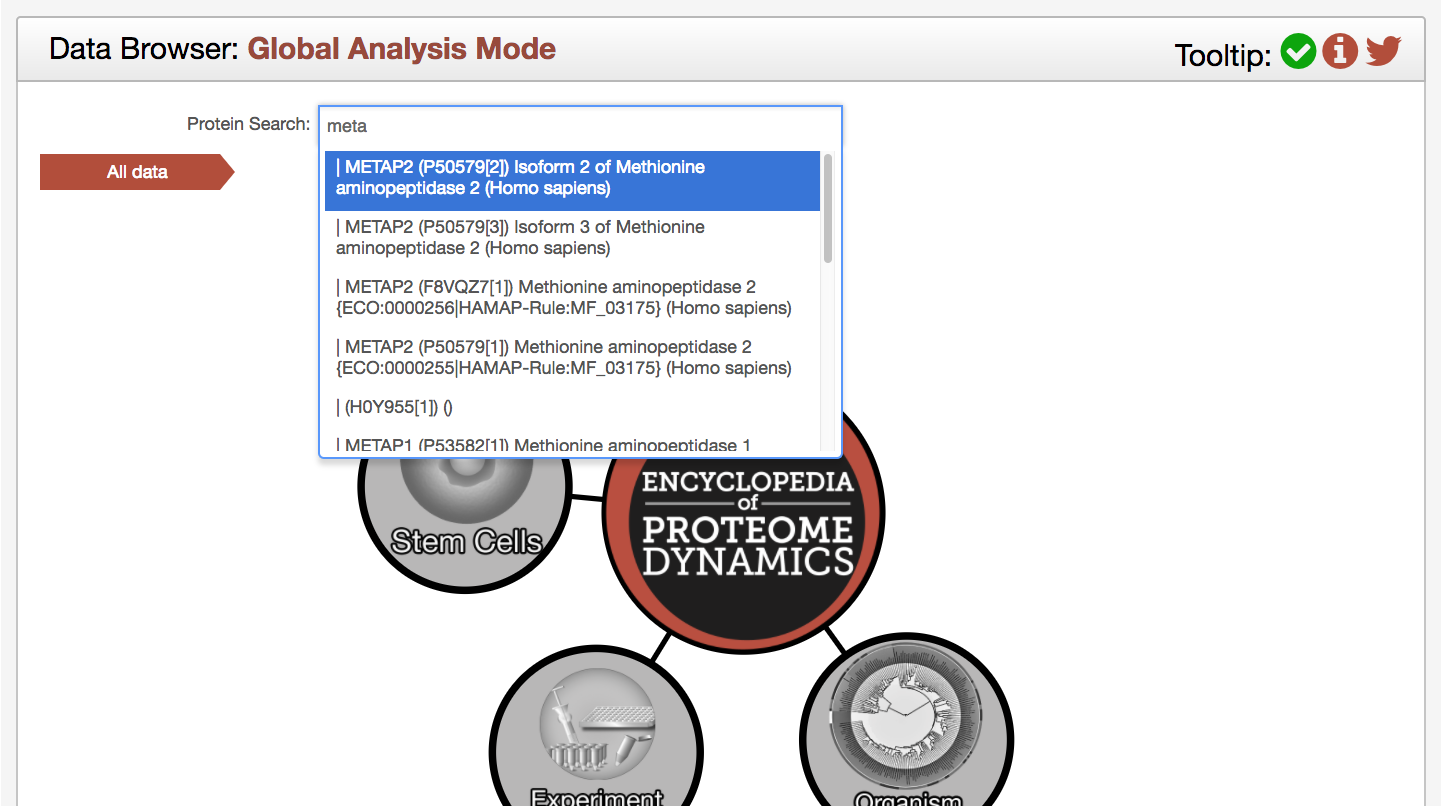


**Supplemental Figure 4:** Protein Search using the Search box

Once the ‘Protein Analysis’ Mode is selected, two new buttons also become available, ‘General Info’ and ‘External Links’. The latter provides convenient links to external, online data resources, including UniProt, PDB and String, already filtered to display information about the protein of interest. The General Information button provides more contextual data for the protein of interest, as shown in Supplemental Figure 5. Each of the Gene Ontology Terms listed in the tab is clickable and directly linked to the Gene Ontology website to obtain additional information about the term.


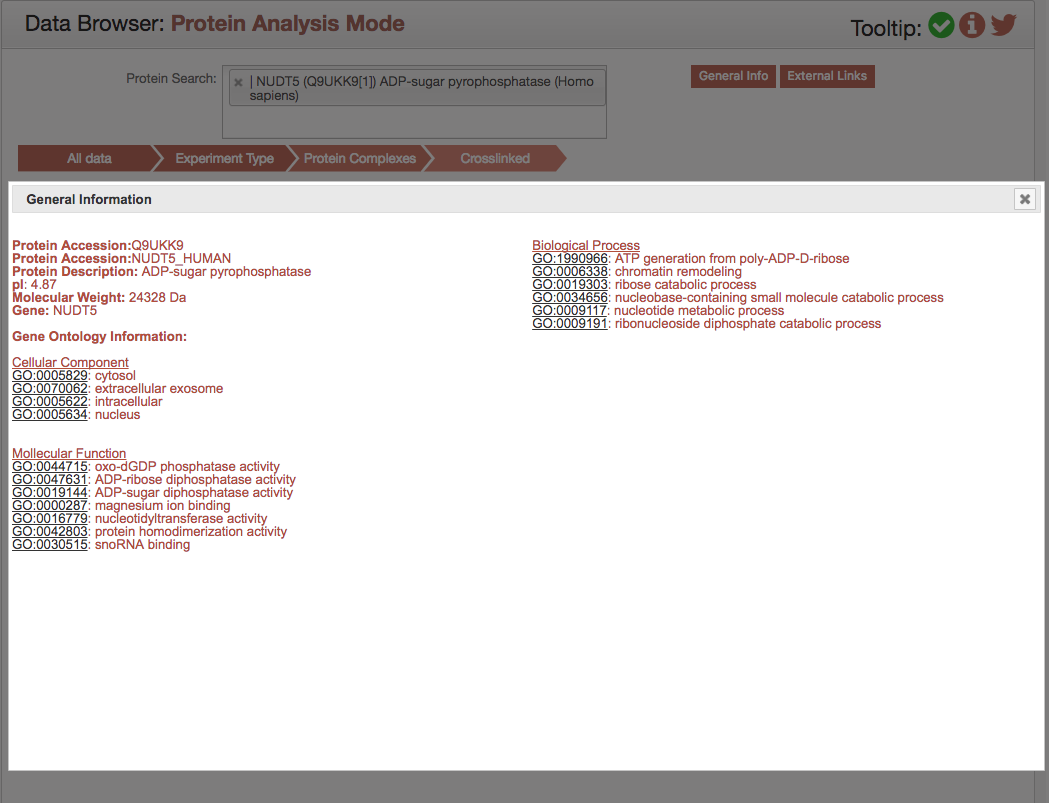


**Supplemental Figure 5:** The General Information interface

Section 2: Line Plots

Line plots in the EPD are also implemented as interactive charts based on D3.js. This functionality is illustrated here using data from a study analysing human Protein Complexes using Size Exclusion Chromatography (SEC)-MS on extracts prepared from U2OS cells that were cross-linked *in vivo* with formaldehyde. The plot shown in Supplemental Figure 6 can be accessed at <https://peptracker.com/epd/analytics/?section_id=10408&protein_id=Q9UKK9> by clicking on the red node with the label, ‘Elution Profile’.

After clicking on the Elution Profile node, a dynamic plot is created that shows the profile for the NUDT5 protein across the respective SEC fractions (Supplemental Figure 6). The Dynamic Plot section is divided into multiple elements; there is a menu bar on the top right with several buttons. Clicking the first button at the left of the menu bar, labelled ‘Download Data Set’, downloads to a user-selected location a comma separated value (.csv) file for all proteins that were detected in the experiment. The ‘Download Plot’ button creates a scalable vector graphic (.svg) file of the plot displayed onscreen and downloads it to the default location. This functionality requires permission for the browser to open pop-ups to be enabled by the user. The ‘Publication’ button provides a direct link to the manuscript where the selected EPD dataset was published and redirects to the relevant ‘Pubmed’ page. The ‘Raw Files’ button provides a direct link to the Pride database (<http://www.ebi.ac.uk/pride/archive/>), where the raw MS files used to generate the displayed protein data are deposited and can be freely downloaded. Finally, the ‘Back to Navigation’ button, located at the right of the menu bar, exits the plot window and returns the user to the interactive navigation window.


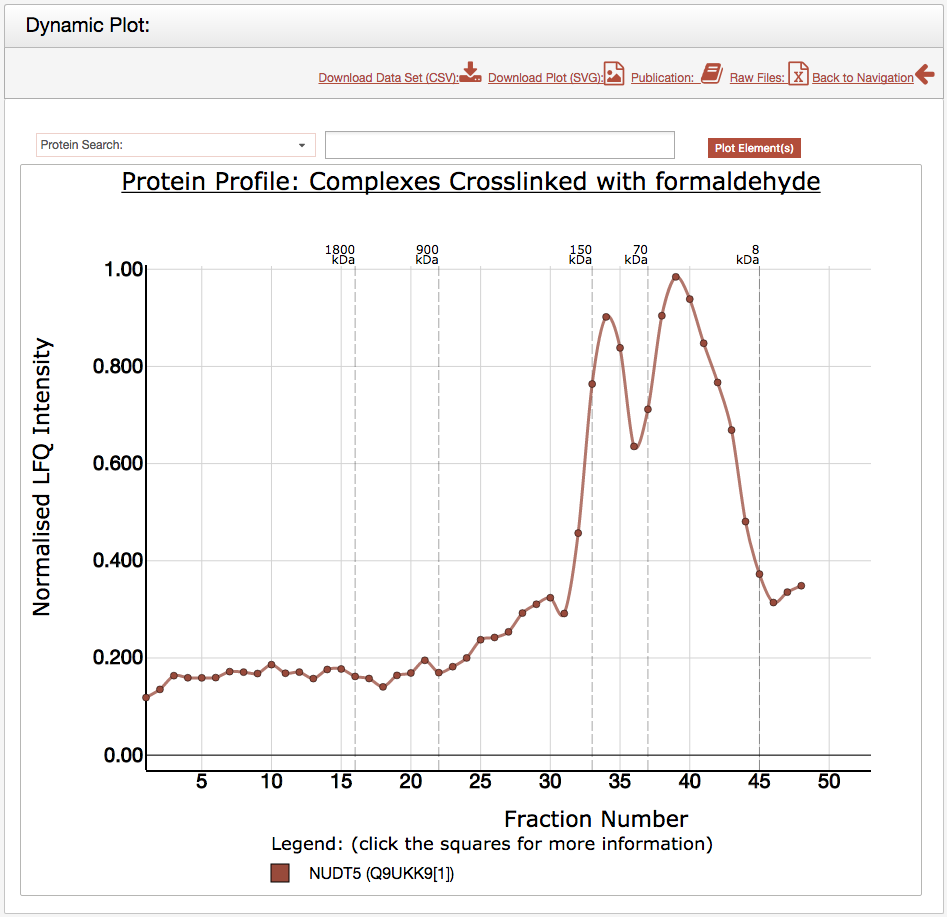


**Supplemental Figure 6:** Protein Profile line plot

The plot section in Supplemental Figure 6 shows a line with dots, corresponding to the protein elution profile across the respective SEC chromatography fractions. Molecular weight markers are labelled on top of the plot to provide context about the SEC separation performance. The dots in the line are interactive elements and they will display a tooltip when the user mouses over them. The tooltip will disappear the moment the mouse is no longer hovering over one of the dots. This tooltip shows additional information about the element, e.g., displaying the number of peptides detected for that protein in the selected fraction and the standard deviation. All the line plots have interactive tooltips that can be moused-over.

Furthermore, there is a legend at the bottom of the plot with a coloured square and a label next to it. This legend uses the colour to represent the selected protein and clicking on the square box will reveal additional functionality, as illustrated in Supplemental Figure 7.


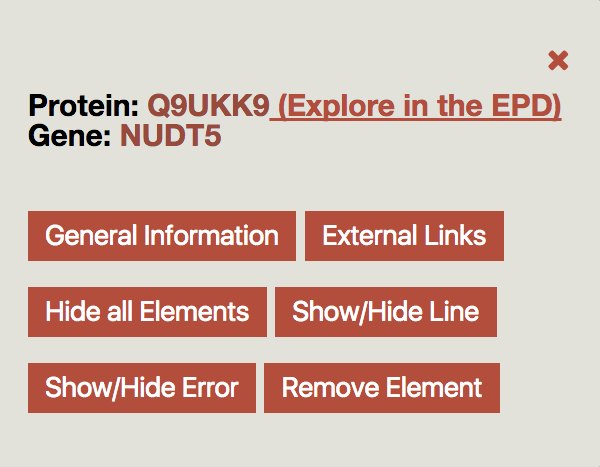


**Supplemental Figure 7:** Legend Tooltip Box

This tooltip provides access to the same ‘General Information’ and ‘External Links’ buttons as found in the navigation page. However, it also has additional elements that provide increased functionality for the line plots. For example, clicking the ‘Hide all Elements’ button will remove all lines from the plot, the ‘Show/Hide Line’ button will toggle the visibility of the selected line only, the ‘Show/Hide Error’ button will toggle the visibility of a coloured ribbon that represents the error measurement for that line and the ‘Remove Element’ button will delete the selected line from the plot entirely.


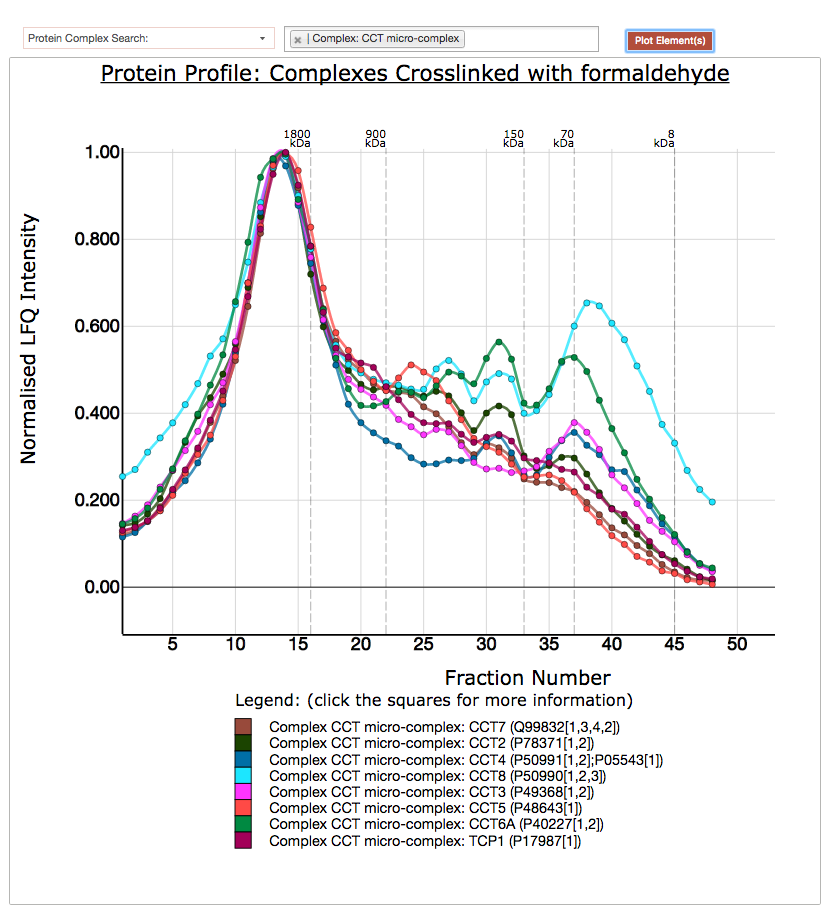


**Supplemental Figure 8:** CORUM Protein Complex search

Above the plot section there is a Search Box, similar to that present in the Navigation section, but with one relevant difference. The Search Box on the plots has additional search options that make use of publically available external data annotations, currently including Gene Ontology (GO) (<http://www.geneontology.org/>), Reactome (<http://reactome.org/>) and CORUM (<http://mips.helmholtz-muenchen.de/corum>). Supplemental Figure 8 exemplifies the search for a specific Protein Complex from CORUM, in this case searching for the Chaperonin Containing T micro-complex within the crosslinked, U2OS cell SEC dataset. This is enabled by selecting ‘Protein Complex Search’ from the Search Box dropdown menu.

Once this search option has been selected, after the user types in more than two characters the Search Dropdown Menu will display all the Protein Complexes from CORUM that (i) have at least one subunit detected in the dataset and (ii) have a name matching the characters that have been typed in the Search Box. In this example, after typing ‘CCT’ in the Search Box and selecting the desired option from the Dropdown Menu, the output shown in Supplemental Figure 8 can be obtained by clicking on the ‘Plot Element(s)’ button at the right of the Search Box.


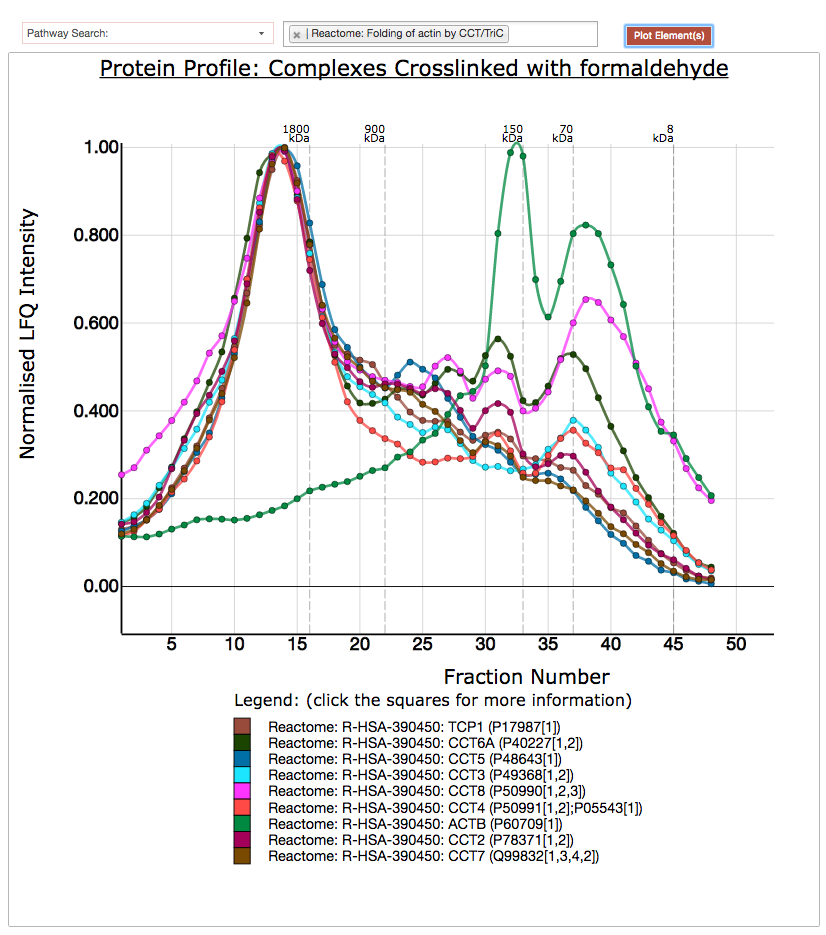


**Supplemental Figure 9:** Reactome Pathway search

A similar procedure can be applied to search the data plot and display elements corresponding to either individual selected proteins of interest, GO terms, or Reactome Pathways. Supplemental Figure 9 shows the result of searching for the term ‘CCT’ in the Search Box when ‘Pathway Search’ has first been selected from the Search Box Dropdown Menu. By selecting ‘Folding of actin by CCT/TriC’ the output displayed in Supplemental Figure 9 is obtained. By comparing Supplemental Figure 9 with the output shown in Supplemental Figure 8, it is apparent that they share 8/9 subunits, with the Reactome Pathway having one additional subunit that shows a different Protein Profile in this dataset.

Section 3: Volcano Plots

The interactivity of plots in the EPD is not limited to line plots. The next example shows a Volcano Plot analysing the change in the proteome of mouse CD8+ cytotoxic lymphocytes after 6 days of treatment with rapamycin. The log_2_ fold change of the protein ratio of the treated population/the control population is shown on the X-axis and the negative log_10_ P-value on the Y-axis.

The plot shown is accessible at <https://peptracker.com/epd/analytics/?section_id=10300> and can be obtained by clicking on the node with a volcano plot icon and the label ‘CTL’.


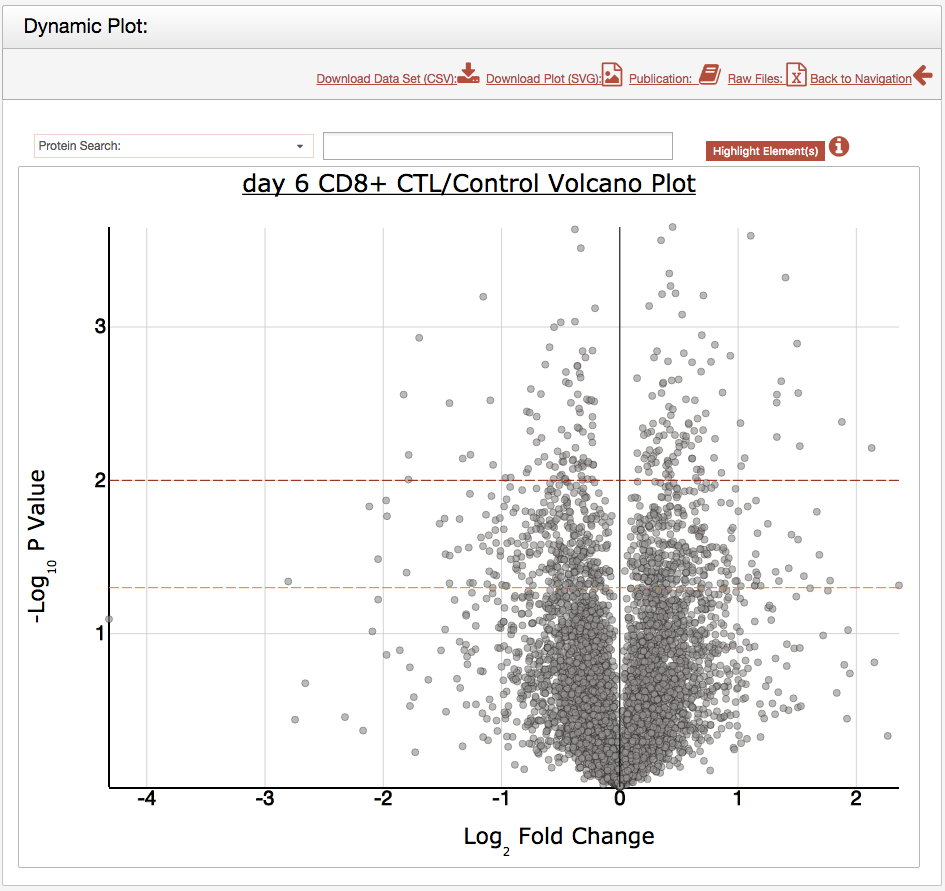


**Supplemental Figure 10:** day 6 CD8+ CTL treated with rapamycin/Control Volcano Plot

Supplemental Figure 10 illustrates a static representation of the volcano plot described previously. The interactivity in Volcano plots differs significantly from lines plots in that it is no longer a ‘mouse-over’ event, but ‘on-click’ instead. This behaviour was employed because the tooltip box that is displayed for selected elements in volcano plots has additional functionality that is not present in the line plots.

Every single dot in the volcano plot in Supplemental Figure 10 represents a protein that was detected in each condition, hence providing a representation of changes occurring at a proteome-wide scale. All the dots in this volcano plot are clickable. The tooltip that is visible after a click event is represented in Supplemental Figure 11.


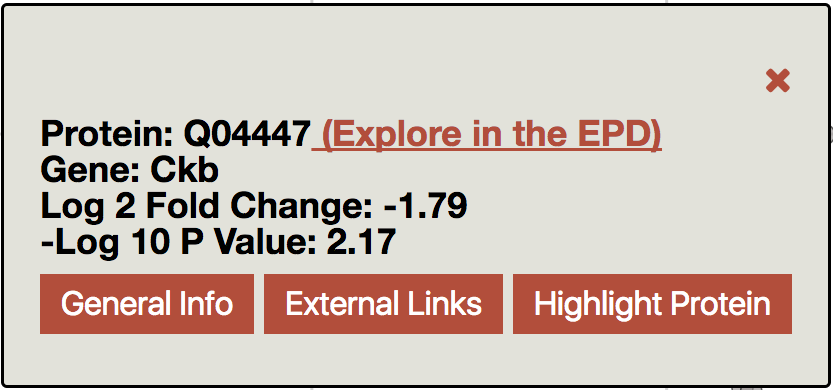


**Supplemental Figure 11:** Volcano Plot Tooltip Box

The first two buttons in the Tooltip Box share the same functionality as provided in the Navigation Section and the Line Plots. However, an additional ‘Highlight Protein’ button allows the user to highlight the selected protein of interest that was clicked, which will be shown as a coloured dot on the plot and will simultaneously integrate this element into the legend, using the same colour. This provides the same functionality that is available when using the Search Box to identify on the plot a specific protein of interest, but allowing a graphical selection for proteins based on their behaviour on the plot. Additionally, the ‘Explore in the EPD’ option in the Tooltip Box provides a rapid and powerful way to obtain more information about a selected protein within the EPD datasets. Clicking on this option will redirect the user to the ‘Experiment Type’ hierarchy, with the protein filter already active and thus only showing the datasets where the selected protein is present.

Moreover, the Volcano Plots share the same expanded search functionality that was described in detail in the previous Line Plot section. Thus, a search for either ‘Protein’, ‘Protein Complex’, ‘Gene Ontology’ or ‘Reactome Pathway’ is also available via the Search Box Dropdown Menu. The only difference is that here the button used to identify the selected element(s) of interest is now labelled as ‘Highlight Element(s)’, since the volcano plot already shows all the elements. Therefore, there is no requirement to ‘plot’ selected elements, just to highlight them with a specific colour. Supplemental Figure 12 illustrates a Volcano Plot after sequential searches for multiple proteins and a Reactome Pathway. The results for each search are highlighted in different colours. As shown with Line Plots, clicking on the coloured boxes in the legend reveals additional functionality.


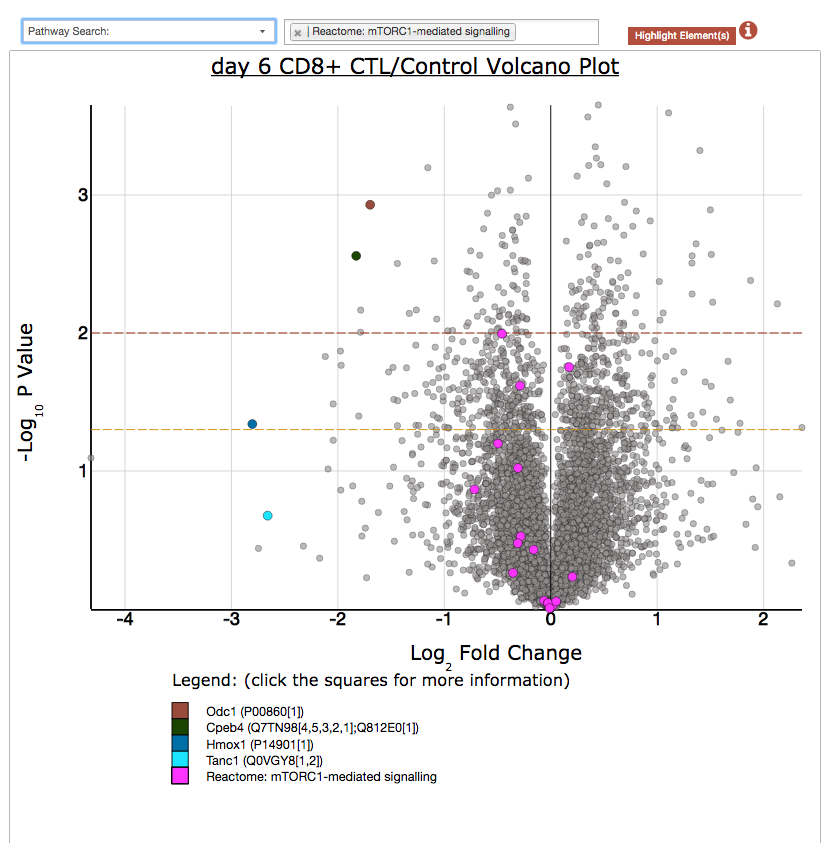


**Supplemental Figure 12:** Volcano Plot showing Protein and Reactome searches

Section 4: Parallel Coordinate Plots

Parallel Coordinate Plots (PCP) are amongst the most complex and dense visualisations within the EPD. They are used to show elements and how they behave throughout multiple different dimensions of analysis. In the example shown in Supplemental Figure 13, the elements correspond to post-translational modifications (PTMs), specifically protein phosphorylation sites. The illustrated data are from a study analysing phosphorylation of proteins in the nematode *Caenorhabditis elegans* isolated from either wild type, or *pig-1* mutant embryos. In this study peptides were analysed both with and without prior affinity chromatography enrichment for phopho-peptides, using TiO_2_ beads.

The example shown in Supplemental Figure 13 is available at <https://peptracker.com/epd/analytics/?section_id=10425> and is accessed by clicking on the node labelled as ‘PTM Sites’. Upon clicking the node the PCP will be displayed. The analysis dimensions are shown as vertical axes and for this example they cover:

- The Amino Acid (either Ser, Thr or Tyr) that has been phosphorylated
- The log_10_ Label Free Quantification (LFQ) intensity for the modified protein
- The log_10_ intensity of the phosphorylation site
- Estimated probability that the phosphorylation site is at the predicted location
- The log_10_ value for the position of the modification within the protein sequence
- The Andromeda Score for the phosphorylation site
- The number of replicates where the specific site was detected

On the current plot, every single phosphorylation site is plotted as a line that intersects all the axes to display the behaviour of the PTM. The sections of the plot that have a darker shade of grey represent a higher percentage of phosphorylation sites sharing the same behaviour, as illustrated in Supplemental Figure 13.


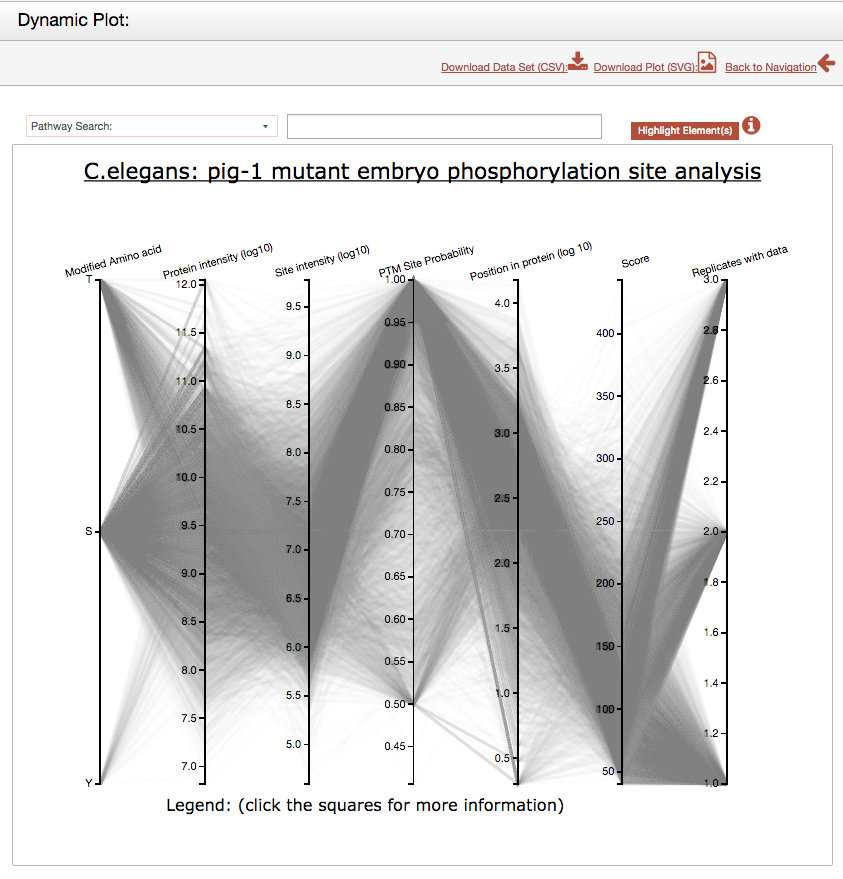


**Supplemental Figure 13:** PCP for *C.elegans* *pig-1* embryo protein phosphorylation sites

The PCP diagrams are also interactive. Thus, clicking on any one of the grey lines will reveal a tooltip box displaying additional information about the site. This is illustrated in Supplemental Figure 14, which shows a Tooltip Box with information on the analysis dimensions that were described above. The Tooltip Buttons at the bottom of the box provide the same functionality that was described previously for other plots.


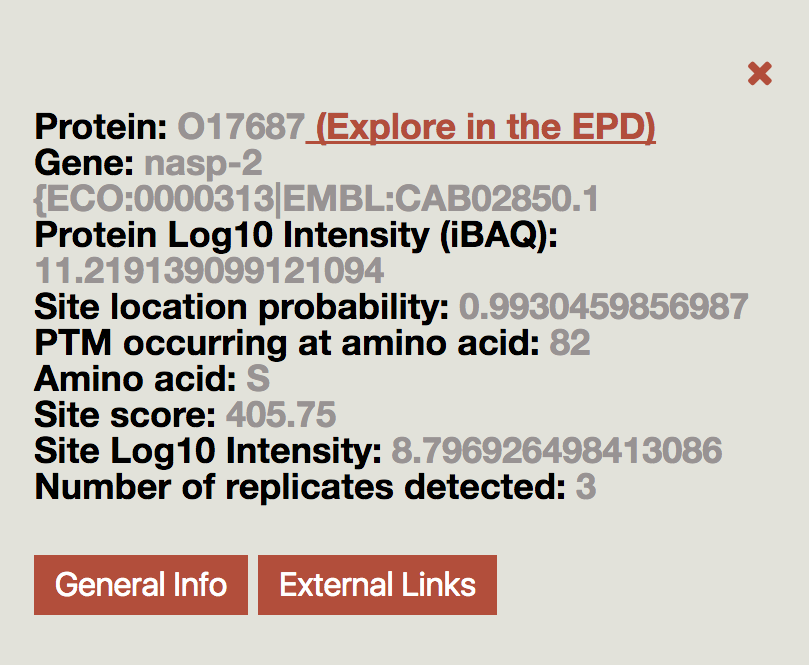


**Supplemental Figure 14:** PCP Tooltip Box

The PCP diagrams also have new dynamic features, allowing filters to be applied dynamically to each of the dimensions of data analysis. By dragging the cursor on the axis of interest a transparent box will appear, highlighting the area of the axis that has been selected. Any values outside of the highlighted section will not be displayed. Users can apply a filter for each dimension of analysis. For example, Supplemental Figure 15 displays this behaviour by filtering phosphorylation sites that were found in all 3 replicates, had the phosphorylation site on a Serine residue and have a site raw intensity value lower than 3,162,277. To remove any of the filters, represented by a transparent box, simply click on any part of the axis of interest that is outside the aforementioned transparent box.


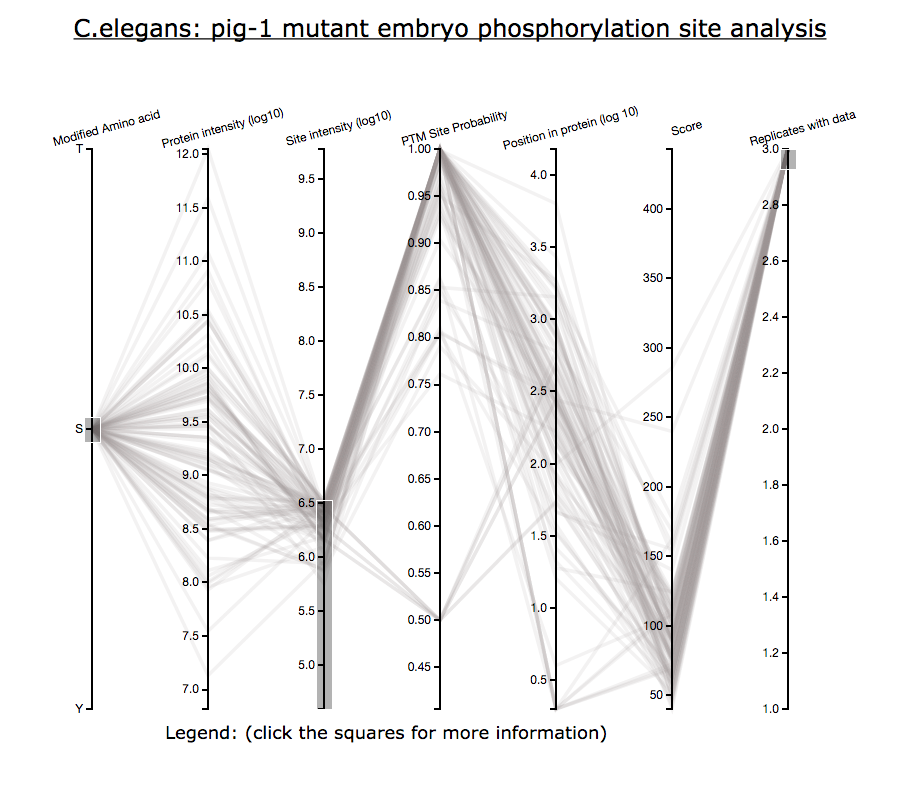


**Supplemental Figure 15:** PCP diagram after applying filters for site, intensity and replicates

In common with the previous plot types described above, PCP diagrams also allow the user to search for either a selected GO Term, Reactome Pathway, Protein, or Protein Complex, by selecting the relevant option from the Search Box Dropdown Menu. This is illustrated in Supplemental Figure 16, where every phosphorylation site for CREB phosphorylation through the activation of Ras are highlighted in brown and a protein search for G5ECG1 highlights them in blue.


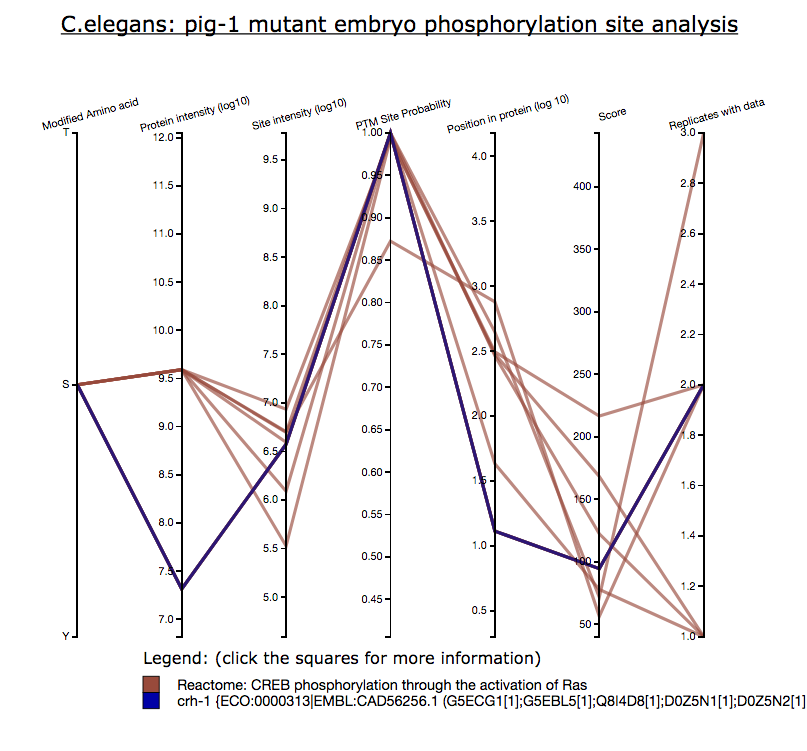


**Supplemental Figure 16:** Protein and Reactome search

The Tooltip Box that is displayed by clicking on one of the elements in the legend provides additional functionality to the user, as illustrated in Supplemental Figure 17. For example, there is a ‘Background Sites’ button to toggle On/Off those phosphorylation sites (referred to as ‘background sites’), which correspond to all of the sites on proteins that are not related to the elements present in the legend. The ‘On/Off Protein Sites’ button will toggle the display of phosphorylation sites for a selected Protein. There are analogous buttons to toggle this display for Protein Complexes, Reactome Pathways and GO terms. The ‘Delete Protein’ button will remove all the sites for the selected element.


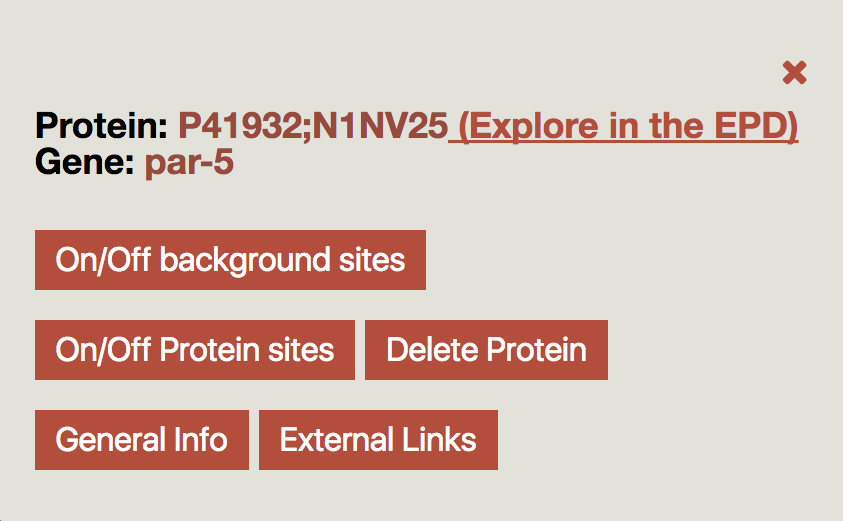


**Supplemental Figure 17:** Tooltip Box for elements selected in PCP Diagram

Section 5: PTM Comparison Scatter Plots

PTM Comparison Scatter Plots differ from other similar plots. They convey information about intensity changes occurring at the PTM site on a protein, for example either during a biological response, or between cells of different genotypes, compared to a control condition. However, they also show on the same plot a comparison of the change in total protein intensity compared to the control condition. As such, the X-axis displays the log_2_ ratio of the condition/control for the protein, while the Y-axis shows the analogous ratio for the PTM site.

The example shown in Supplemental Figure 18 is available at <https://peptracker.com/epd/analytics/?section_id=10425> and can be obtained by clicking on the node ‘PTM Site Analysis’. The orange dotted line passing through the origin shows where the protein ratio and the PTM ratio are equal. After calculating the mean deviation of phospho sites from the identity, the orange area represents all values that are less than 1 standard deviation away from the identity line, while the pink area represents elements where the value of the ratio is between 1 and 2 standard deviations. All elements outside the pink area have values more than 2 standard deviations away from the identity line.


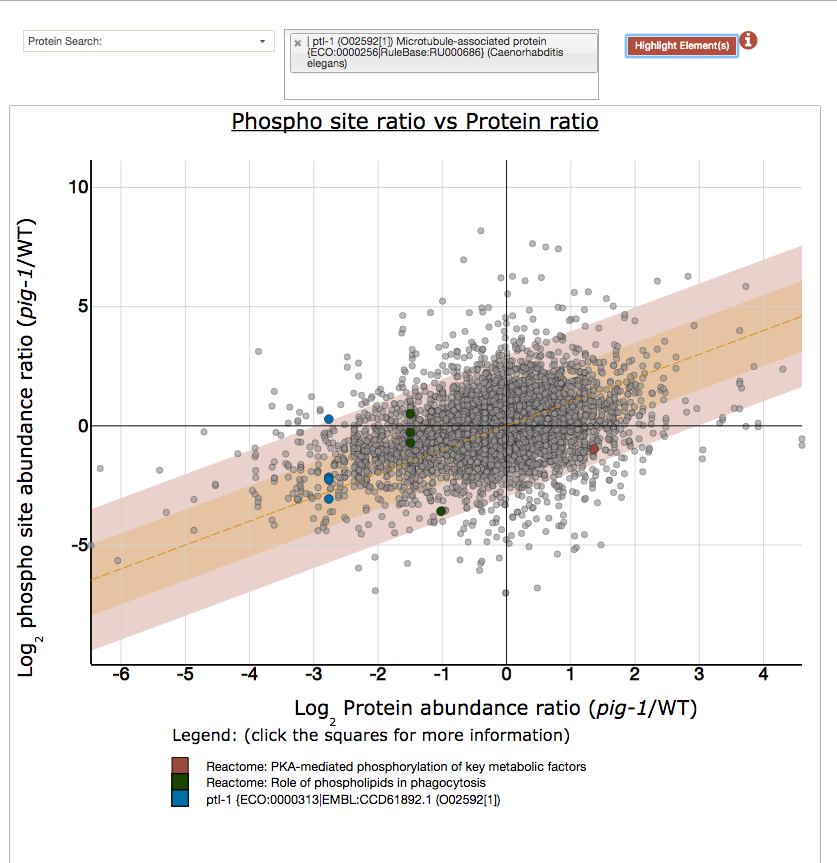


**Supplemental Figure 18:** Protein and Reactome search in PTM Comparison Scatter Plots

In common with the other types of plot described above, the PTM Comparison Scatter Plots also provide users with the same options to search for and display specific element(s) of interest, including Proteins, Protein Complexes, GO terms and Reactome Pathways. Supplemental Figure 18 illustrates a PTM Comparison Scatter Plot displaying the results of sequential searches for two Reactome Pathways and one specific protein. Note that PTM sites that are located in the same protein form a vertical line. This occurs because while the site ratio will change, altering values on the Y-axis, they are constrained to the same position on the X-axis since the protein they belong to will have the same abundance ratio in all cases.

Section 6: Heatmaps and Force Diagrams

To display information about data clustering from multiple experiments, the EPD uses a combination of force diagrams and heatmaps. For example, the force diagram illustrated in Supplemental Figure 19 shows data on Protein Complexes, derived from co-fractionation experiments, which relates clusters of proteins with similar chromatographic behaviour. Each cluster is represented as a dark central node, surrounded by a ring of coloured nodes. Each coloured node in each ring represents an individual protein that clustered with the other proteins in that ring. The dark central node denotes the identity of that cluster. Clicking on a coloured node will open a tooltip box displaying additional information about the selected protein and it will simultaneously fade out all the other clusters and focus on the selected one. Supplemental Figure 19 illustrates an example of such a Force Diagram. It is available at <https://peptracker.com/epd/analytics/?section_id=10408> and can be accessed by clicking on the node ‘Clusters’.


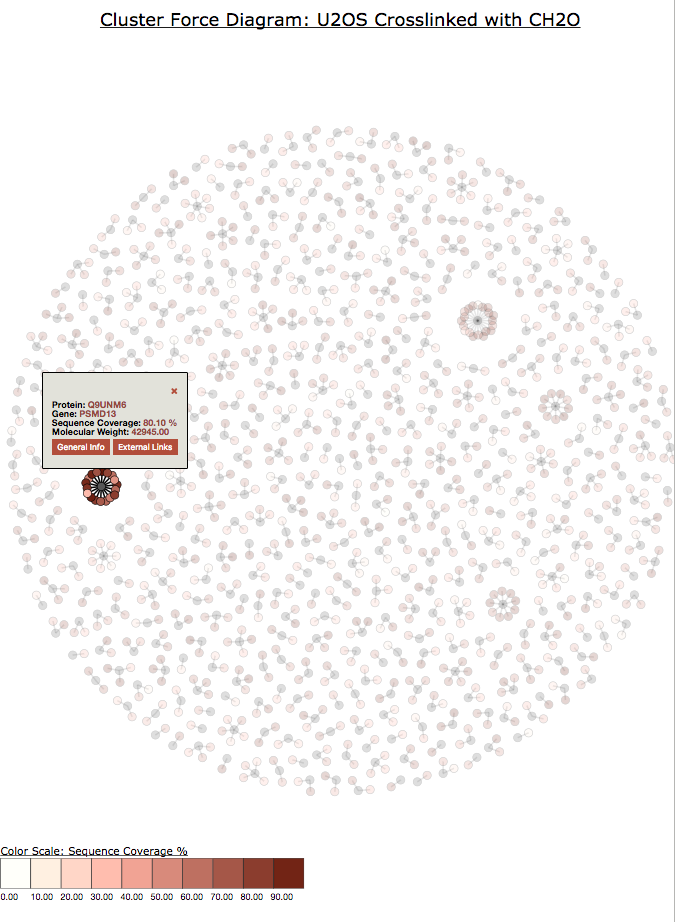


**Supplemental Figure 19:** Protein Clusters represented as a force diagram

The colour scheme in this case represents the sequence coverage that was obtained for each of the proteins in the cluster. The force diagram displays a different tooltip box when you click on one of the dark nodes in the middle of each cluster. This tooltip will show a button labelled ‘Plot Cluster Elements’. When this button is clicked, a search is performed for all the protein subunits that clustered together and these are displayed in a line plot, as shown in Supplemental Figure 20. The force diagrams are available on the Global Analysis mode only.


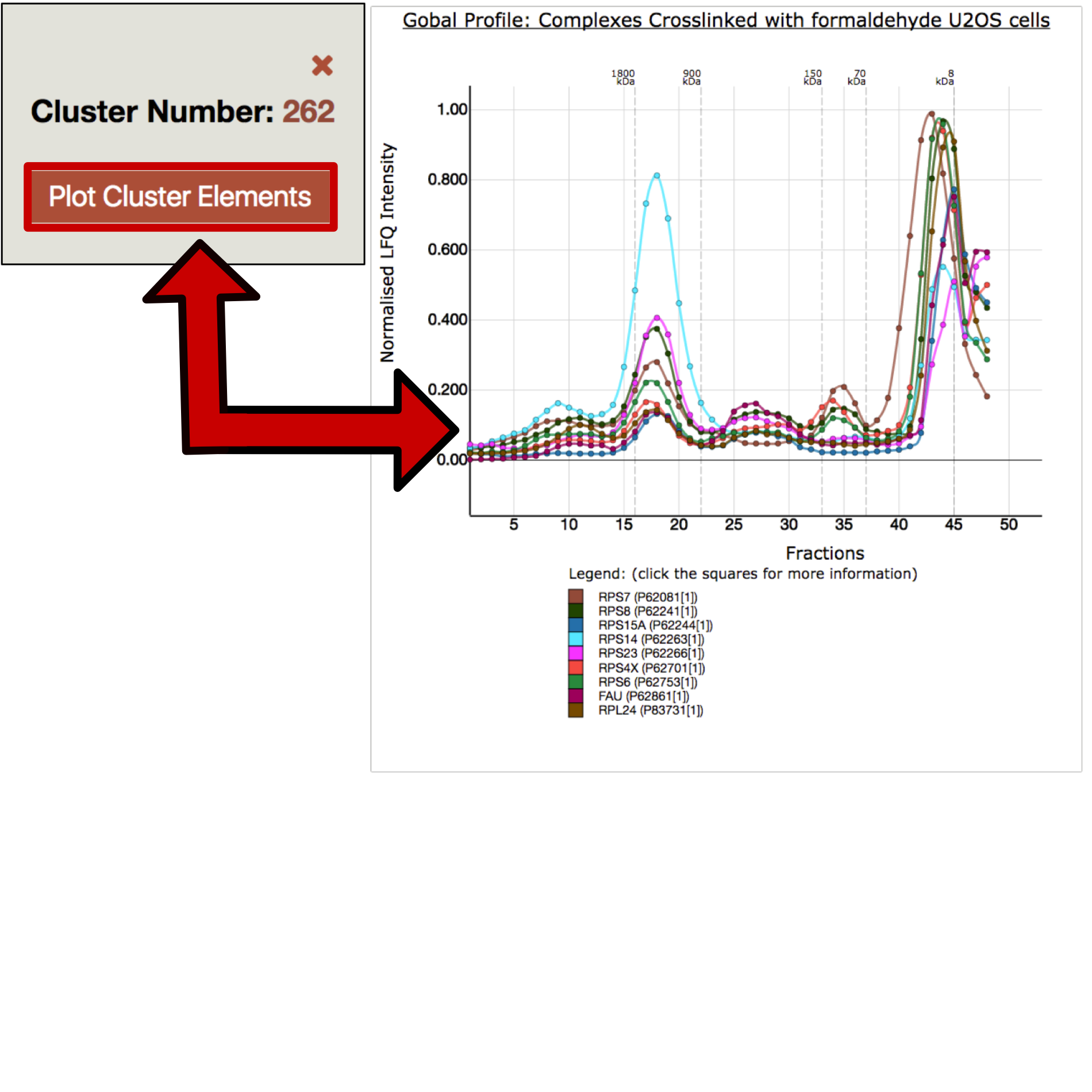


**Supplemental Figure 20:** Force diagram tooltip and the ‘Plot Cluster Elements’ functionality

When searching for a specific protein via the Search Box, if the selected element was present in any protein cluster, a heatmap node will appear as one of the end nodes for the dataset. Supplemental Figure 21 demonstrates an example of this functionality, available at <https://peptracker.com/epd/analytics/?section_id=10408&protein_id=O00232> and accessible by clicking on the node ‘Heatmap’. Heatmaps will show the profile of the clustered elements, e.g. either across the chromatographic fractions for interactome experiments, or across different time points for kinetics of response experiments. Supplemental Figure 21 shows proteins that clustered together with protein PSMD12 (O00232) and their Feature Scaled LFQ profile across the different fractions of a SEC experiment. All the squares on the heatmap have a mouse-over interactive behaviour.


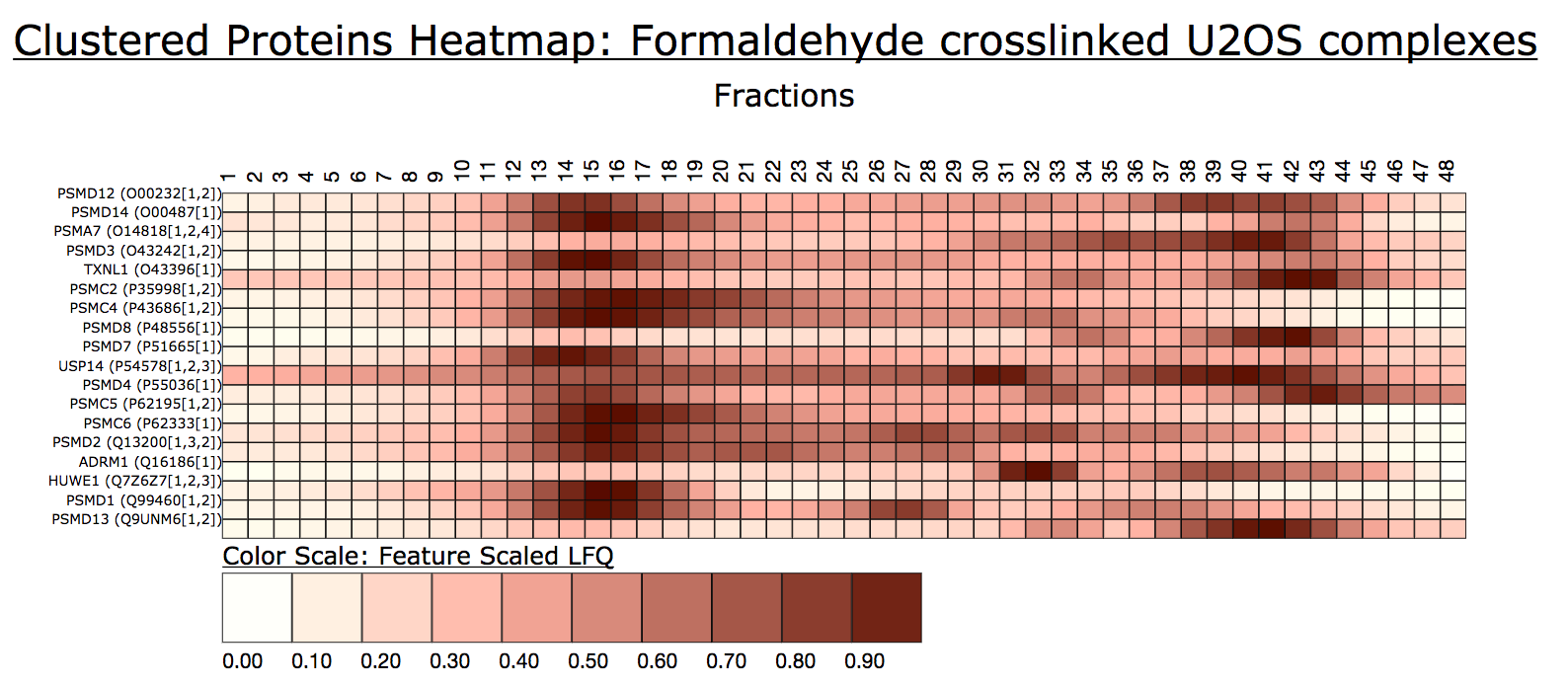


**Supplemental Figure 21:** Heatmap for proteins clustered with PSMD12

Section 7: Bar plots, box plots and histograms

Bar plots, Box plots and Histograms in the EPD all share a mouse-over interactive behaviour, but do not currently have available the extra search options to select and highlight either specific Proteins, GO Terms, Reactome Pathways, or Protein Complexes. Bar and Box Plots are used interchangeably, depending on the analysis mode that is active on the EPD. If it is operating in Global Analysis mode then Box plots will be present, while in Protein Analysis mode a Bar plot will be displayed. This is illustrated here using data from an analysis of proteome variation upon cell cycle arrest in human myeloid leukemia NB4 cells.

First, we show the global analysis mode used with this dataset, which is available at <https://peptracker.com/epd/analytics/?section_id=10209> and can be accessed by clicking on the Node with a box plot image and the label ‘Fractions’. Upon clicking on this node a Box and Whiskers plot (Box plot) will be displayed, as illustrated in Supplemental Figure 22.


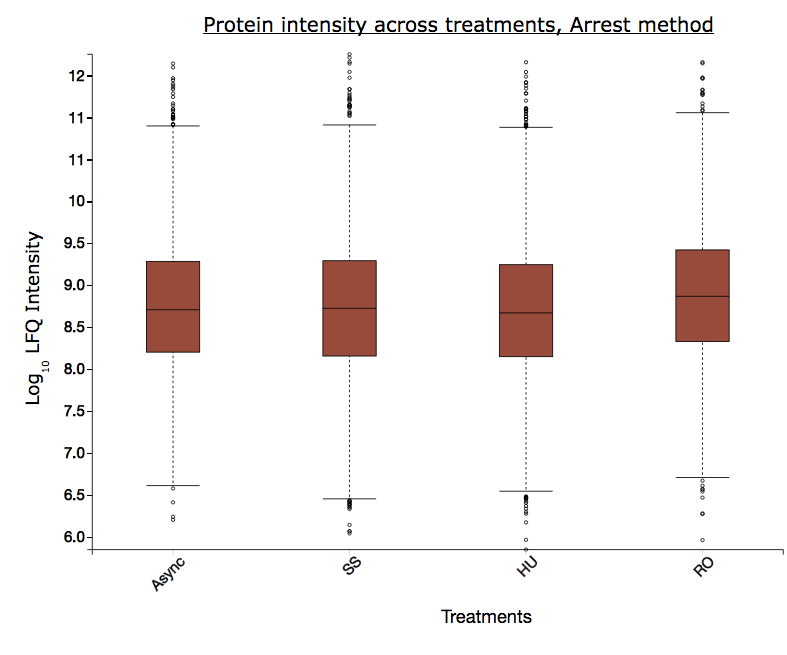


**Supplemental Figure 22:** Box plot of Log_10_ Label Free Protein Quantification in myeloid leukemia cells

Box plots provide useful information about how data measurements are distributed. The bottom of each of the red boxes shows the first quartile, the horizontal dark solid line inside the boxes represents the median and the top of each box represents the third quartile. This box is interactive. Placing the cursor over it will display a Tooltip showing the numeric values of these 3 elements. Elements that are represented as circles outside the areas covered by the whiskers are the outlier data points for that distribution.

To analyse how a specific protein behaves across the parameter being studied, in the example shown this parameter is cell cycle phases, then going into Protein Analysis mode is required. The example illustrated in Supplemental Figure 23 is available at <https://peptracker.com/epd/analytics/?section_id=10209&protein_id=Q9NZJ9>. This link is the equivalent of searching for protein Q9NZJ9 (NUDT4) from the previous link. The Box Plot image has now been replaced with a Bar Plot, labelled ‘Fraction’.


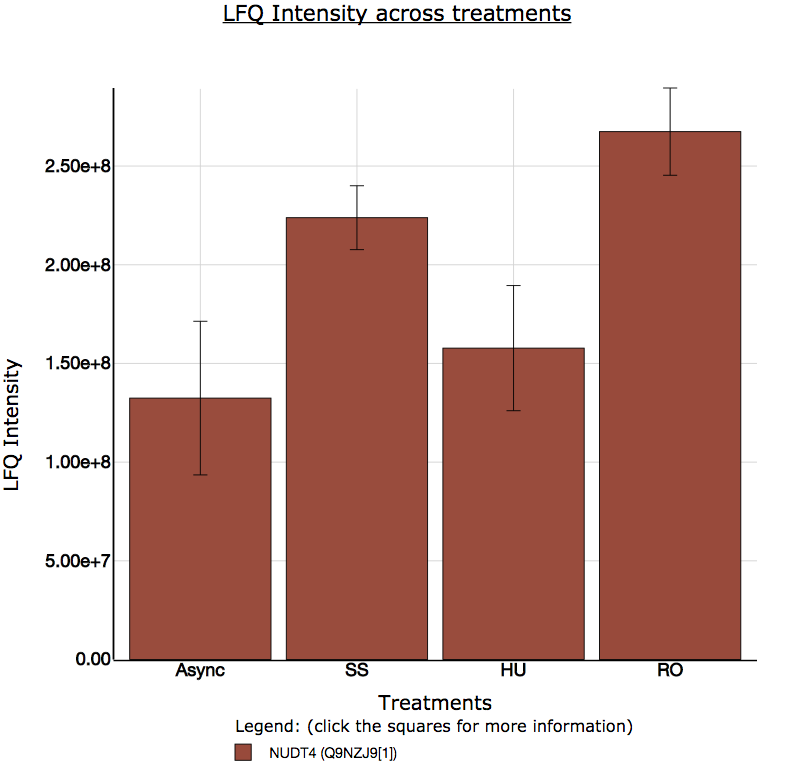


**Supplemental Figure 23:** Bar plot of Label Free Quantification in myeloid leukemia cells for protein NUDT4

Supplemental Figure 23 illustrates the effect of clicking on the Bar plot icon. Each bar represents the Label Free Quantification (LFQ) intensity for the protein NUDT4 for each of the treatments that arrest the cell cycle. Each of the bars is interactive. Placing the cursor over a bar will reveal a Tooltip showing the LFQ intensity value and the treatment label. Additionally, error bars are overlaid on top of the bars to display the error and a legend section is displayed below for the protein of interest. The coloured square shown in the legend has a click event that will display a Tooltip Box providing links to the ‘General Information’ and ‘External links’ information for that protein.

The final plot in this family of visualisations is the histogram. The example shown is the log_10_ iBAQ intensity for all proteins detected in asynchronously growing myeloid leukemia cells. It is available at <https://peptracker.com/epd/analytics/?section_id=10327&protein_id=Q9NZJ9> and can be accessed by clicking on the node with a Histogram icon and the label, ‘Protein’.

Histograms can be accessed in both Global and Protein Analysis modes. As with Bar and Box plots, they have a mouse-over interactive behaviour. Placing the cursor over any bar will display a Tooltip showing the range of values the bar represents and the number of elements that are present in that range. If the histogram is accessed in Protein Analysis mode, as illustrated in Supplemental Figure 24, then a legend will appear at the bottom of the histogram. The legend has the same functionality as described in the Bar Plot section above.


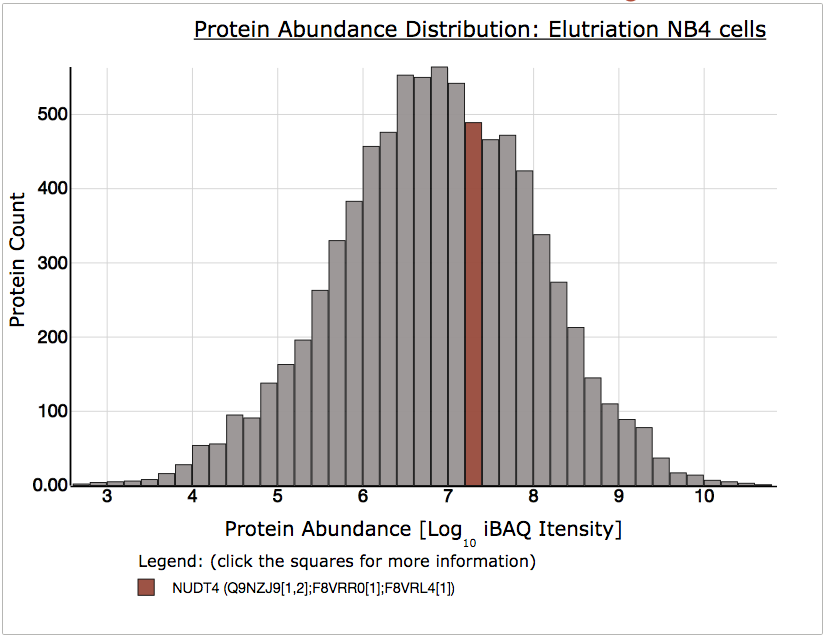


**Supplemental Figure 24:** Histogram showing the log_10_ intensity Based Absolute Quantification in myeloid leukemia cells with the corresponding bin for protein NUDT4 highlighted.

Section 8: Bubble plots

Bubble plots are visualisations where every protein that is detected in a dataset is represented as a circle (bubble). The circles are organised in a spiral shape, where proteins with the highest quantified intensity are located on the outer edge and the lowest intensity elements are in the middle of the spiral. Bubble plots are available for display in both log_10_ scale as well as untransformed intensity. This can be selected by clicking on the drop-down menu labelled ‘Abundance Measure’ and selecting the desired option.

The example shown in Supplemental Figure 25 uses a log_10_ iBAQ scale. It is available at <https://peptracker.com/epd/analytics/?section_id=40100>. It can be accessed by clicking on the node with the label, ‘All’.


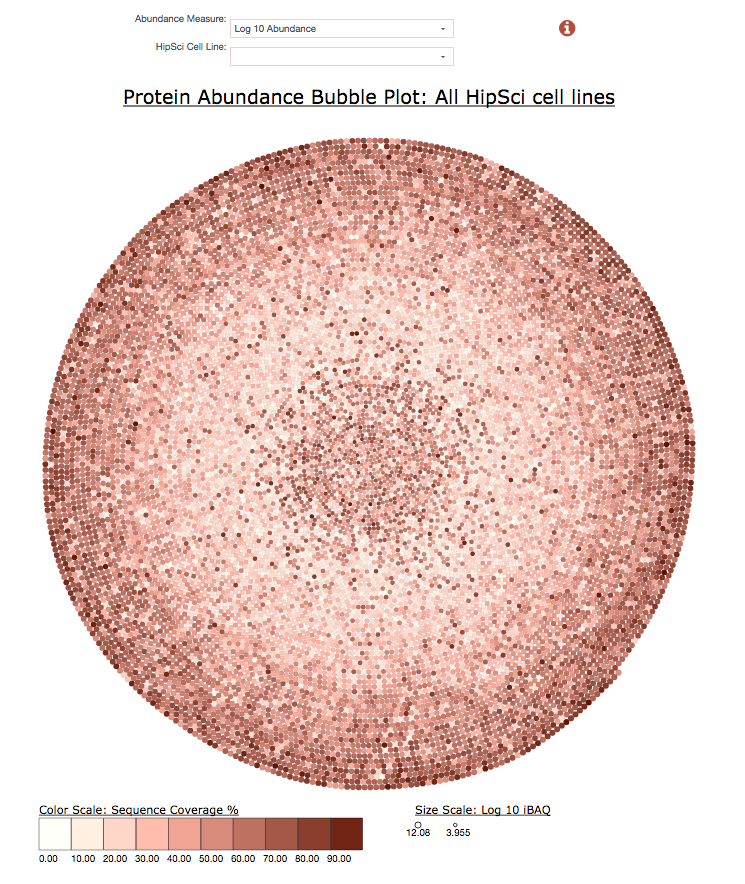


**Supplemental Figure 25:**Bubble plot showing the log_10_ iBAQ of all proteins detected for the human induced pluripotent stem cells.

Every circle represented in the plot is a protein. All circles have interactive on-click behaviour, similar to that present in the volcano plots. The size of the circles is determined by the log_10_ iBAQ value and their colour indicates the percentage sequence coverage. The log_10_  scale shown in this example is ideal to observe patterns of coverage across the population.

However, to observe the changes in raw abundance the log_10_ scale is not ideal. Clicking on the ‘Abundance Measure’ drop-down and selecting ‘Abundance’ will create a new plot based on the intensity measure without a log_10_ transformation. The example shown in Supplemental Figure 26 is the same node as in Supplemental Figure 25, but after clicking on the ‘Abundance Measure’ drop down menu and selecting ‘Abundance’, instead of ‘Log 10 Abundance’.


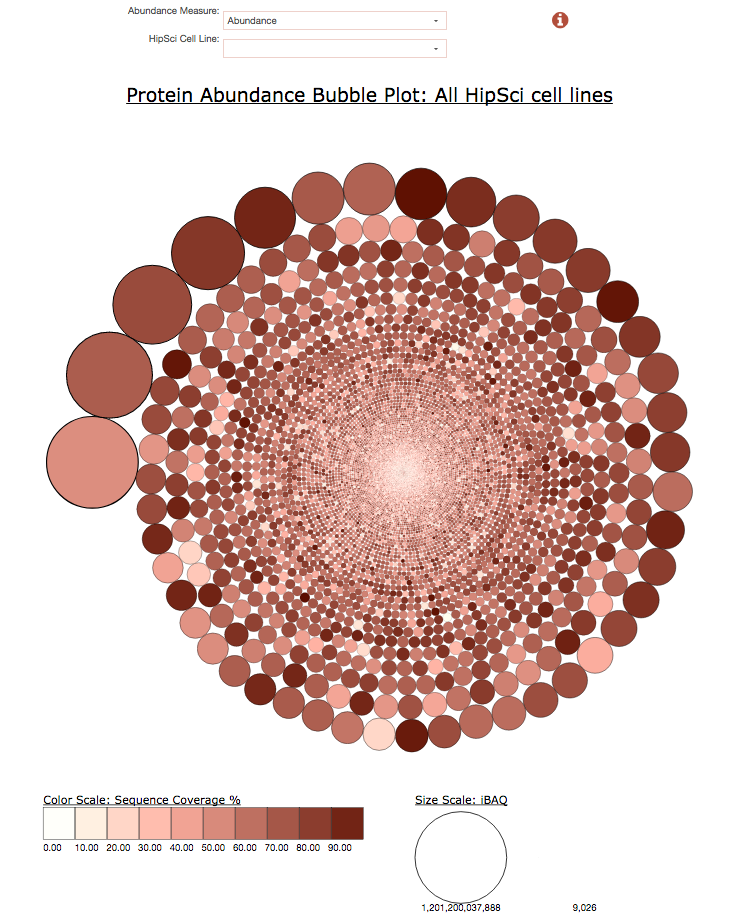


**Supplemental Figure 26:** Bubble plot showing the iBAQ of all proteins detected for the human induced pluripotent stem cells.

In this case the extreme differences in the diameters of proteins in the centre vs outside of the circle reflect the large dynamic range in protein expression levels typical of cell proteomes.

Section 9: Report an issue

A very important focus within the EPD is user feedback and experience. To aid continual improvements in functionality, every page contained within the EPD has a blue button labelled ‘Report issue’ on the middle right section of the browser. This button allows users to report any behaviour that the user feels needs to be improved, or is not working as intended.


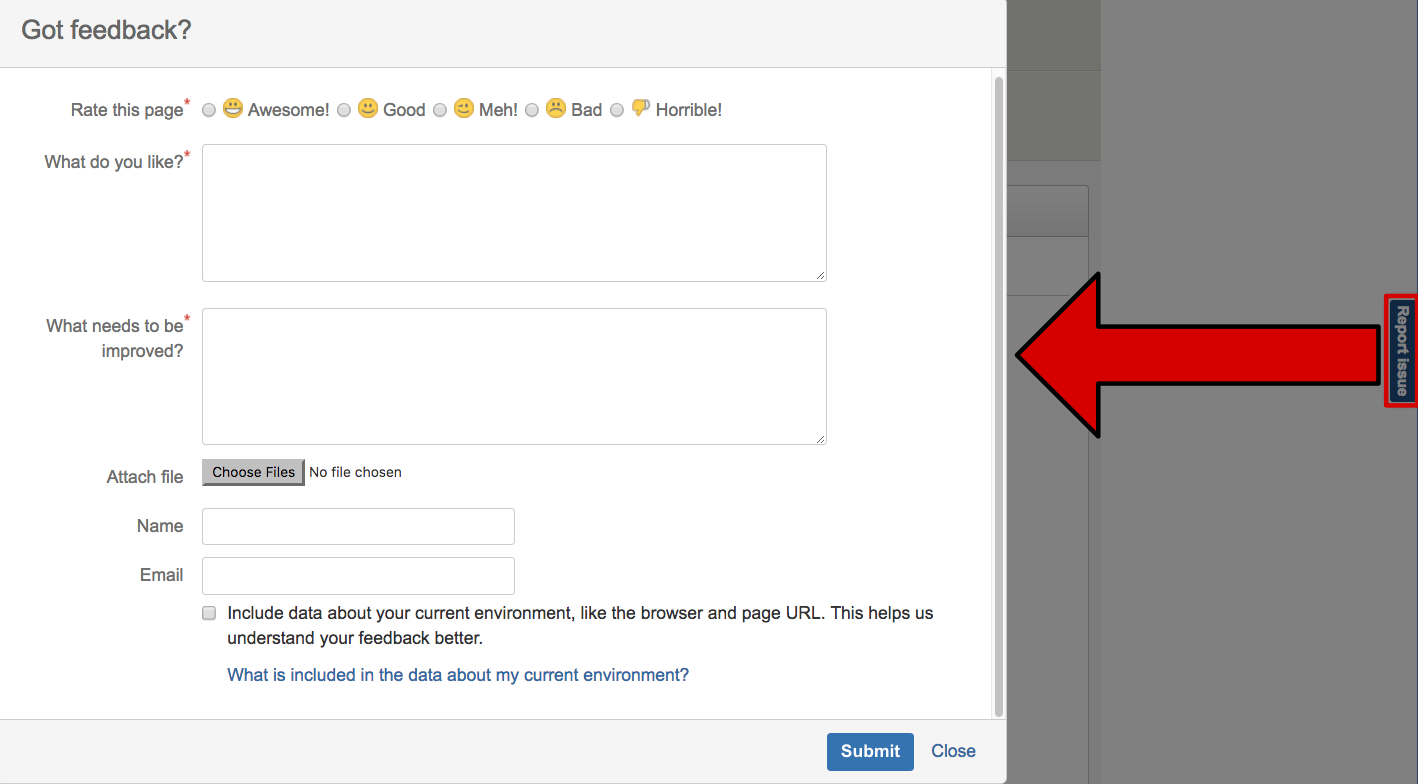


**Supplemental Figure 27:** Report issue functionality

Supplemental Figure 27 illustrates the form that is displayed after clicking on the ‘Report issue’ button. Users can describe the issue they encountered, attaching a file to describe the issue (screenshots are very popular) as well as attaching environment information by selecting the last checkbox. By clicking on submit, the issue is sent to our development team.
